# RNA-Seq analysis reveals an essential role of tyrosine metabolism pathway in response to root-rot infection in *Gerbera hybrida*

**DOI:** 10.1371/journal.pone.0223519

**Published:** 2019-10-23

**Authors:** Nigarish Munir, Chunzhen Cheng, Chaoshui Xia, Xuming Xu, Muhammad Azher Nawaz, Junaid Iftikhar, Yukun Chen, Yuling Lin, Zhongxiong Lai

**Affiliations:** 1 Institute of Horticultural Biotechnology, Fujian Agriculture and Forestry University, Fuzhou, China; 2 Sanming Academy of Agricultural Sciences, Sanming, Fujian, China; 3 Department of Horticulture, College of Agriculture, University of Sargodha, Sargodha, Pakistan; 4 Fujian Provincial Key Labortary of Plant Functional Biology, College of Horticulture, Fujian Agriculture and Forestry University, Fuzhou, China; Key Laboratory of Horticultural Plant Biology (MOE), CHINA

## Abstract

*Gerbera hybrida* is one of the top five cut flowers across the world, it is host for the root rot causing parasite called *Phytophthora cryptogea*. In this study, plantlets of healthy and root-rot pathogen-infected *G*. *hybrida* were used as plant materials for transcriptome analyis using high-throughput Illumina sequencing technique. A total 108,135 unigenes were generated with an average length of 727 nt and N50 equal to 1274 nt out of which 611 genes were identified as DEGs by DESeq analyses. Among DEGs, 228 genes were up-regulated and 383 were down-regulated. Through this annotated data and Kyoto encyclopedia of genes and genomes (KEGG), molecular interaction network, transcripts accompanying with tyrosine metabolism, phenylalanine, tyrosine, and tryptophan biosynthesis, phenylpropanoid and flavonoid biosynthesis, and plant hormone signal transduction pathways were thoroughly observed considering expression pattern. The involvement of DEGs in tyrosine metabolism pathway was validated by real-time qPCR. We found that genes related with tyrosine metabolism were activated and up-regulated against stress response. The expression of *GhTAT*, *GhAAT*, *GhHPD*, *GhHGD* and *GhFAH* genes was significantly increased in the leaves and petioles at four and six dpi (days post inoculation) as compared with control. The study predicts the gene sequences responsible for the tyrosine metabolism pathway and its responses against root-rot resistance in gerbera plant. In future, identification of such genes is necessary for the better understanding of rot resistance mechanism and to develop a root rot resistance strategy for ornamental plants.

## Introduction

*Gerbera hybrida* (2n = 2x = 50) is a perennial herbaceous flower planted as cut flower, pot flower and garden plants [1]. It has a unique attractive flower arrangement and a wide range of colors. This species belongs to sunflower family Asteraceae and it is native to Africa, Tropical Asia, South America and Madagascar [2]. Recently gerbera has gained an attention as a model plant for developmental and biological studies.

Due to distinctive capitulum inflorescence and floral stem, gerbera has a great aesthetic value. Unlike other species having solitary inflorescences such as roses, it has three different types of florets that are compacted at the same receptacle [3]. Marginal ray florets are zygomorphic and strongly ligulate with fused and showy petals. Its inner disc florets are hermaphrodites; whereas marginal ray florets and trans florets are female and non-functional staminodes. Disc florets consist of fertile pollens whereas ray florets have elongated ligulate petals and aborted stamens [2]. The corolla is condensed in disc flowers in the direction of the middle of inflorescence and the symmetry vary to the radial from bilateral. Unlike other model flowers, *Arabidopsis thaliana*, *Petunia hybrid* and *Antirrhinum majus*, its inferior ovary matures under the curls of flowery organs [4, 5, 6]. The commercially cultivated varieties have been developed by the artificial crossing of South African species of *Gerbera jamesonii* and *Gerbera viridifolia* and those are heterozygous.

To study of floral growth and development in Asteraceae flowers, gerbera has proved a model plant. Its variation in disc and ray florets pattering, flower color and high levels of secondary metabolites make it putative model flower for biosynthetic research [7]. Gerbera plant produces some secondary metabolites that help defend against insects and microbial attacks [8]. Root rot is a very common disease in gerbera (*Gerbera jamesonii* L. and *Gerbera hybrida*), and the primary causes of root rot are *Fusarium oxysporum* and *Phytophthora cryptogea*. Plants are frequently affected by *Phytophthora cryptogea*, and it is more pathogenic compared with *Fusarium oxysporum* for gerbera plants. *Phtopyhora creptogea* has approximately 150 host plant species, among those 40 species belongs to flowering plants [9].

The biochemical mechanism of root rot in plants is very complex. The genomics and proteomics proved helpful in revealing the mechanisms and root cause of many diseases and controlling these infestations. However, the genomic resources are rarely available for ornamental crops; the transcriptome RNA sequencing provides an excellent opportunity to study the ornamental plants. Considering the large genome size of Gerbera, the use of sequencing is helpful that reduces the cost and decreases the processing time, significantly. It also helps to focus on specific genes for the establishment of genomic resources. Transcriptome RNA sequencing (RNA-Seq) has recently emerged as a wide and accurate tool for expression pattern analysis of genes, because of its extensive genomic range, higher reproducibility and the superior evaluation for expression levels, particularly under the situations where genomic means are not common and the chance of heterozygosity exists [10]. RNA-Seq is utilized in transcript annotation for the identification of novel transcript regions in plant species [2, 11, 12]. In *Gerbera hybrida*, the transcriptome analysis was built up from the high-throughput sequencing of expressed genes. Expression profiling of different tissues was also developed at different flowers stages using microarray methodology. However, the sequencing technology restricted this profiling, and up till now, 16,994 gerbera (EST) expressed sequence tags have been generated. From gerbera, there are 487 nucleotide sequences, 339 proteins and 17,000 ESTs in NCBI public databases [2]. Nevertheless, although the molecular components of phytohormones have been comprehensively examined in different organs of model and non-model plants, the metabolism of amino acids under biotic stress is not studied in gerbera. Only a couple of segments have been identified to describe the metabolism and plant hormones signal transduction.

This study focus on the identification and characterization of differentially expressed genes of healthy and diseased plants using utilizing NGS sequencing. Through the configuration of reads, assembled by *de novo* assembly, we identified the genes that can be utilized for further genetic investigations. The overall goal of this study was the identification of defense-related genes that are differentially expressed in various pathways when gerbera plants are exposed to *Phytophthora cryptogea*. For the validation of DEGs, real-time quantitative PCR was performed for the genes involved in tyrosine metabolism pathway. This helps to recognize the role of these genes in defense mechanism against pathogen infection in gerbera plants. Transcriptomes are examined by gene annotation and anticipated the candidate genes that are involved in disease resistance pathways particularly for root rot. Identification of DEGs involved in disease defense-related pathways helps study the role of gene in gerbera against root rot. These DEGs provide evidence and can be utilized in gerbera crop improvement program for root rot resistance.

## Results

### RNA-Seq using Illumina platform and assembly of unigenes in gerbera

A total of 113,980,180 reads were produced by means of Illumina high-throughput sequencing TM 2000. Subsequent to the separation of low-quality reads, 110,371,574 clean reads were selected. Among these, 57,026,700 and 53,344874 clean reads of healthy and diseased gerbera plant sample were obtained, respectively (Table 1). Among all reads, the Q_20_ percentage was 97% and GC content for the libraries was 45%. These short reads were *de novo* accumulated into 141,972 unigenes by matched-end joining and gap-filling using Trinity programming. The normal length of these transcripts was 727 with N50 corresponding 1274 nt, extending from 200 nt to > 2000 nt. Subsequently, the contigs were combined until neither one of the ends was expanded. An aggregate of 108,135 unigenes were achieved with an average length of 602 nt and a concluding N50 equal to 937 nt (Table 2). The size allocations showed that the length of 10,671 unigenes was greater than 1,000 nt. Statistics of the size of transcript and unigenes are provided in S1 Fig (supplementary material). The assembly software Trinity is excellent for reconstruction and it is highly sensitive; it can cover full-length transcripts through a broad range of expression levels than other *de novo* transcripts assemblers.

**Table 1:** Summary of *G*. *hybrida* sequence analysis.

Sample	Raw Reads	Clean Reads	Clean Bases	Error (%)	Q20 (%)
**C**	59001654	57026700	8.55 Gb	0.01	97.38
**Dis.**	54978526	53344874	8 Gb	0.01	97.81
**Total**	113980180	110371574	16.55 Gb		

C: Control; Dis: root-rot disease

**Table 2:** Summary of transcriptome of *G*. *hybrida*.

Sample	Total number	Total nucleotides	mean length	N50
**Total number of unigenes**	108135	65063492	602	937
**Total number of transcripts**	141972	103194053	727	1274

### Functional annotation and pathways of unigenes in gerbera

**Fig 1:**
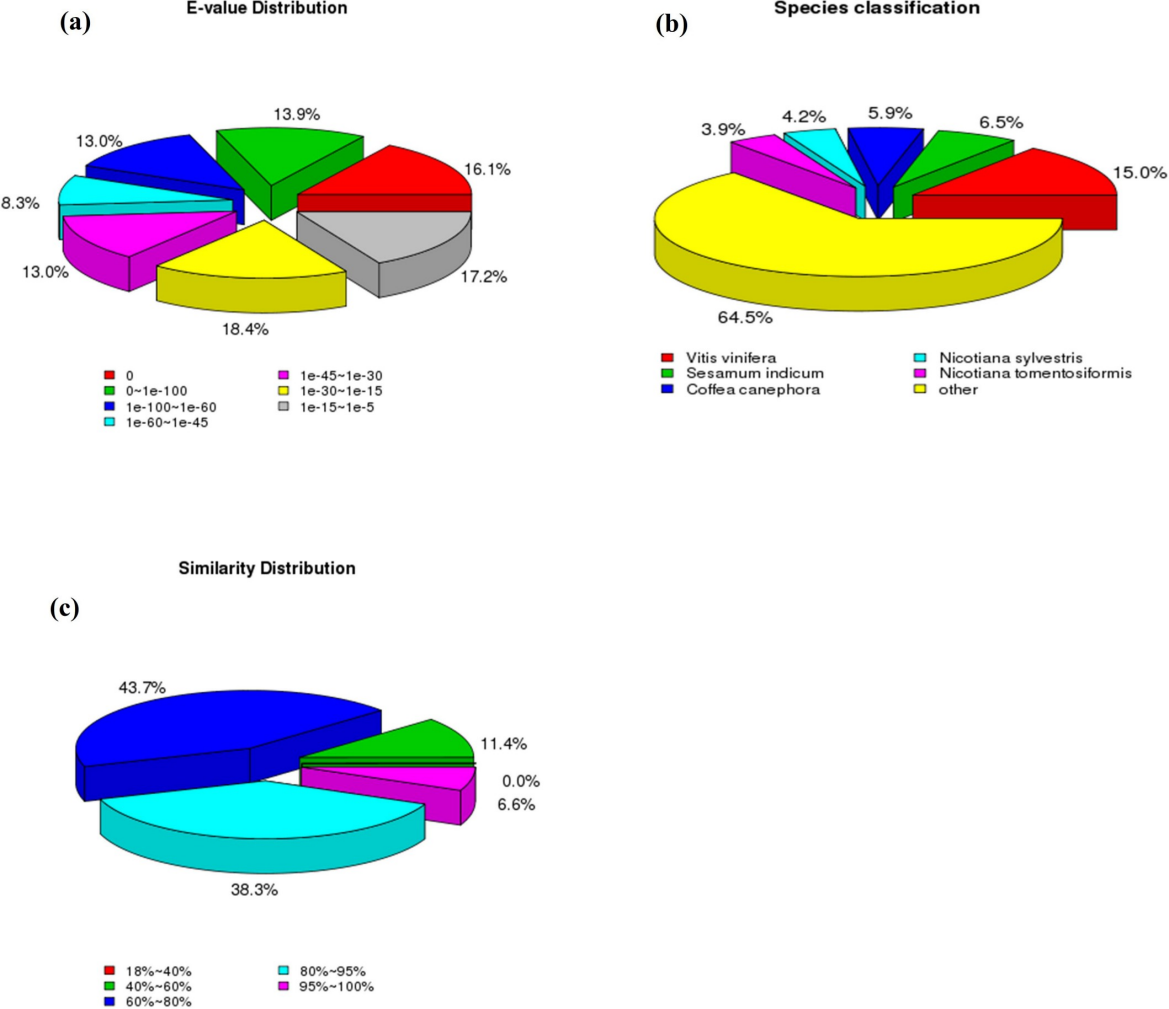
Nr categorization of gerbera Unigenes. (a) E-value distribution (b) Similarity distribution (c) Species classification.

Subsequently excluding repetitive and short size sequences, 38,922 unigenes (35.99% of all cleaned unigenes) were annotated by BLASTX against Nr through cut-off E-value 1e^-5^ for the identification of putative mRNA. The E-value distribution of unigenes in Nr database demonstrated that 30% of the unigenes were strongly similar (smaller than 1e^-100^) however, enduring 70% of sequences extended from 1e^-5^ to 1e^-100^ (Fig 1A). The ratio of corresponding distribution showed that 44.90% of the sequences have over 80% resemblance and remaining 55.10% of the sequences had 18% to 80% similarity (Fig 1B). The specie allocations for the best match for each sequence are presented in Fig 1C. We assumed that the gerbera transcriptome analysis has a close relation with family Solanaceae becuase of their nearby phylogenic association. The outcomes revealed that sequences of gerbera indicated 64.50% comparability with *Solanum tuberosum*, *Populus trichocarpa*, *Citrus clementine* and *Ricinus communis* (Fig 1C). About 15% sequences showed similarity with *Vitis vinifera* (Vitaceae family), 6.5% with *Sesamum indicum* (Pedaliaceae family), and 5.9% have similarity with *Coffea canephora* (Rubiaceae family).

**Fig 2:**
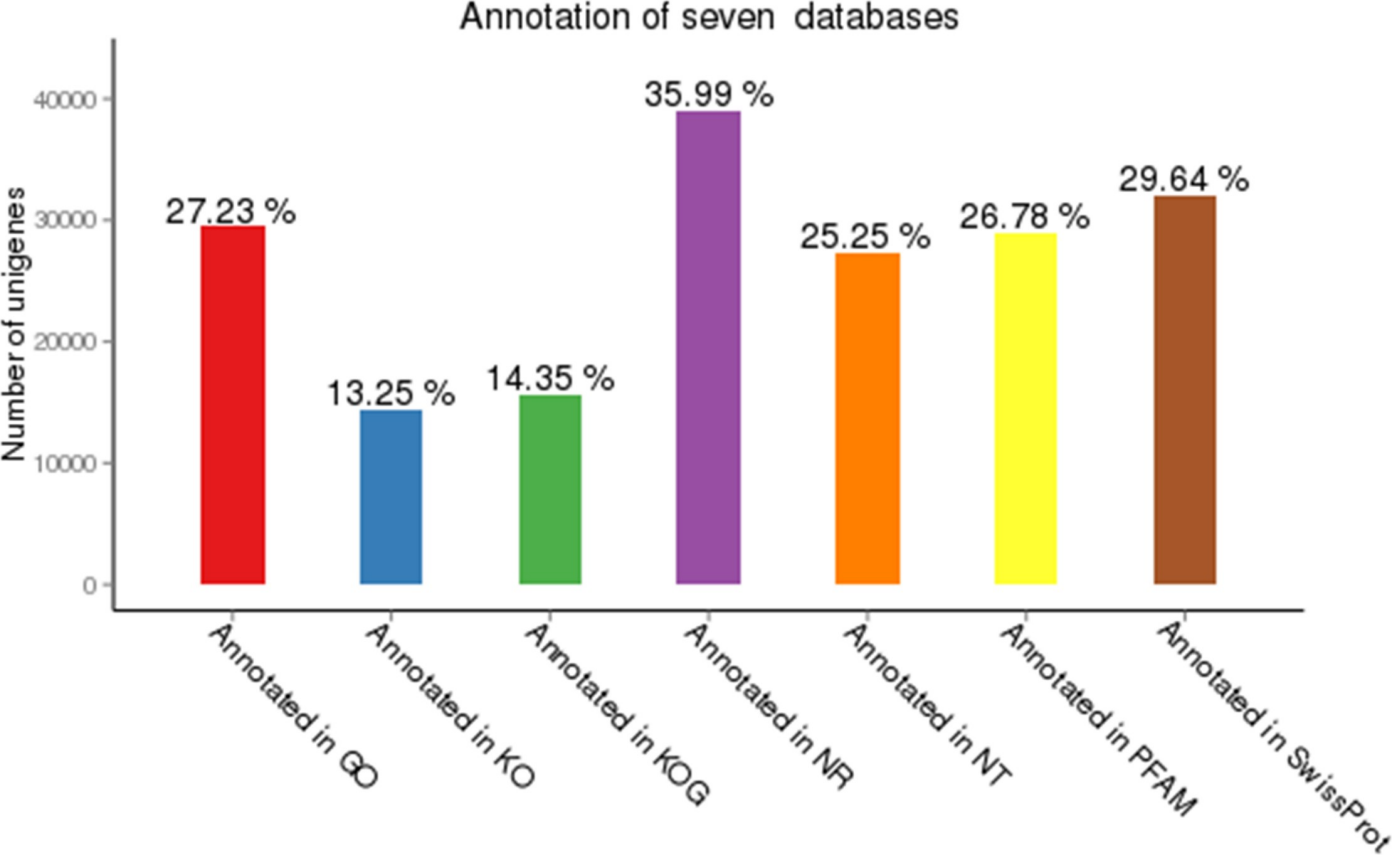
Summary of gene annotation against seven databases in gerbera.

In addition, unigenes were exposed to NR, NT, KO, Swiss Port, Pfam, GO and KOG databases using BLAST analysis (E-value <0.00001). In addition to this, 27,314 unigenes (25.25% of all prepared unigenes) were annotated using BLASTN in contrast with Nt under indistinguishable parameters. Unigenes were likewise adjusted to further databases comprising 32,062 genes (29.64% of all cleaned unigenes) in Swiss-Prot database, 28,964 genes (26.78% of all cleaned unigenes) in Pfam, 29,451 genes (27.23% of all cleaned unigenes) in GO and 15,528 genes (47.89% of all cleaned unigenes) were annotated in KOG database. In total 51,796 (47.89% of all cleaned unigenes) unigenes were effectively annotated from atleast one of the Nr, Nt, KO, SwissProt, GO, COG, KEGG and Pfam databases (Fig 2 and S1 Table).

The distribution of gene functions in GO (Gene Ontology) was grouped into biological processes, cellular component and molecular function. Total annotated genes were 29,451, among each term cellular (16,369 genes), metabolic (15,485 genes) and single–organism process (11,891 genes); cell (8,489 genes), cell part (8,482 genes), organelle (5,588 genes)”; binding (15,516 genes), catalytic activity (12,600 genes) and transporter activity (1,922 genes)” were dominant subcategories (S2 Fig).

Similarly, 15,528 putative proteins marked by COG were categorized into 25 molecular families, and the uppermost classification was General function prediction (2,769 genes) followed by post-translation modification, protein turnover, chaperones (1,992 genes), translation, ribosomal structure and biogenesis (1,433 genes), and signal transduction mechanisms (1,394 genes) (S3 Fig).

Unigenes were also annotated against KEGG database for understanding advanced-level utilities and functions of the biological structure. In this way, 14,335 unigenes were assigned to 19 pathways. By representing the enzyme commission (EC) numbers in contrast to the KEGG database, numerous transcripts were discovered that are involved in metabolism and signal transduction pathways. Metabolism was the most significant category, a substantial number of genes were related to carbohydrate metabolism (1,422 genes) and amino acid metabolism (852 gens) (S4 Fig).

### Exploration of differentially expressed genes and KEGG pathway enhancement examination

**Fig 3:**
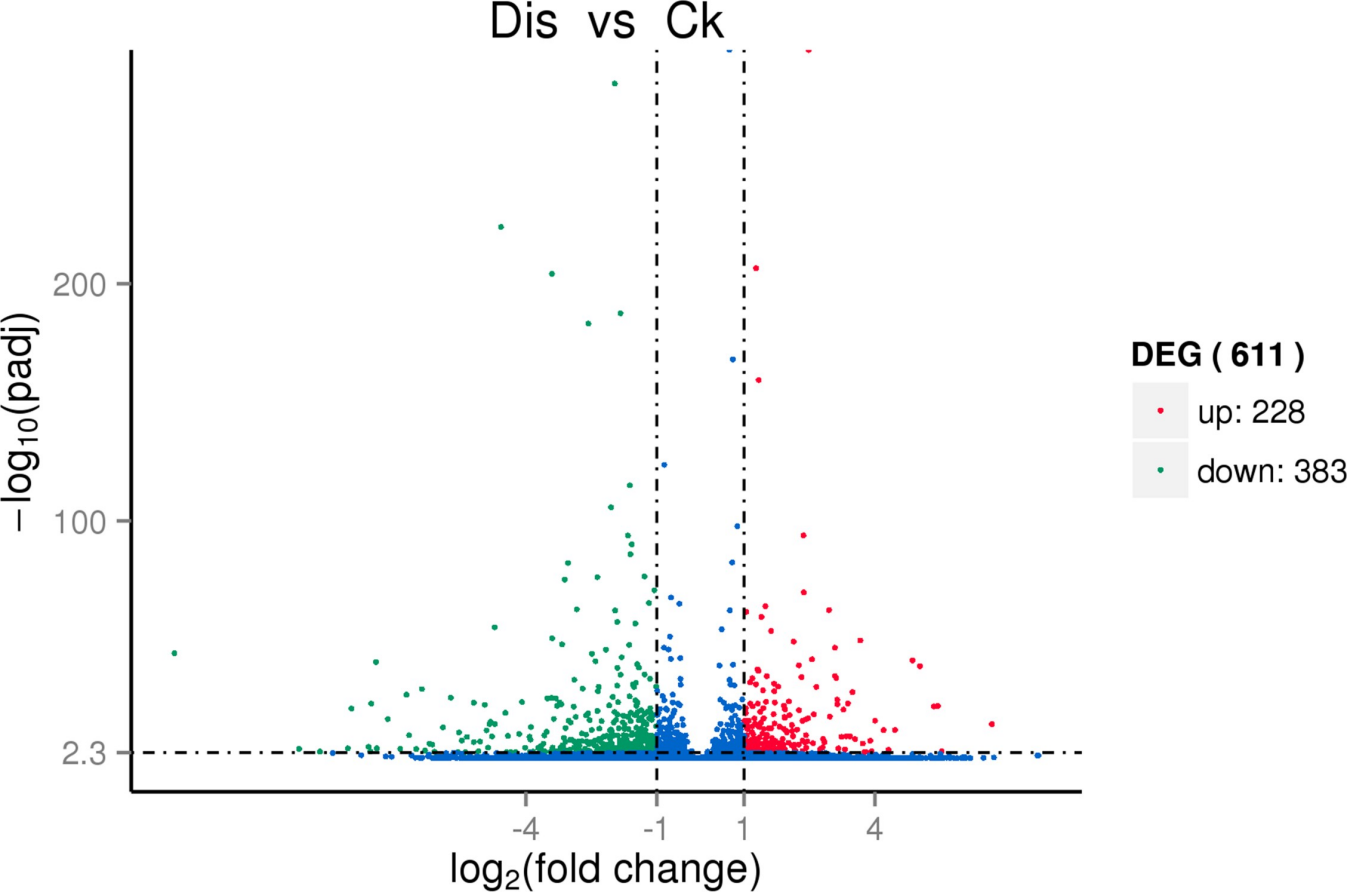
Volcano plot demonstrating differential expressed genes among the Dis (disease) and C (control) gerbera samples. The x-axis is the genes expression change of different sample in a different experiment, and the y-axis is the *p*-value after normalized. The bigger -log10 (q-value) represents more significant differences.

We found 611 unigenes differentially expressed amongst two samples by comparing the expression levels. A total of 228 genes were up-regulated and 383 genes were down-regulated in gerbera in response to the infection of *Phytophothora cryptogea* (Fig 3). For instance, the GO terms single organism metabolic process (GO: 0044710), carbohydrate metabolic process (GO: 0005975), catalytic activity (GO: 0003824), and cell wall (GO: 0005618), were enriched in analysis of DEGs. Interesting, among these DEGs, there were several up-and down-regulated genes that are involved in metabolic and catalytic activities. The functional distribution of up-and down-regulated DEGs with corrected *p*-value < 0.05 are provoided in S2 Table.

Functional annotation demonstrated that root rot disease apprantly (corrected *p* value < 0.05) influenced 15 biological process, 15 molecular functions and 5 cell component metabolic pathways. The root rot disease largely influenced the following biological process in gerbera: single-organism metabolic process, oxidation-reduction process and carbohydrate metabolic process; the affected molecular function includes catalytic activity and oxidoreductase activity; the affected cellular component includes cell wall and cell periphery (S5 Fig). The up and down regulated DEGs that involved in resistance against root-rot disease with their functional annotation, Log_2_ fold change (FC), and *P* value adjusted are listed in Table 3.

**Table 3:** Differentially expressed genes in gerbera plant that are involved in disease resistance against root-rot disease.

Gene ID	Dis read-count	Ck read-count	FC	*p*. value	*q*. value.	Functional annotation
c54071_g1	56.825651	6.0599569	3.2292	7.75E-12	1.22E-09	Tyrosine aminotransferace
c55425_g1	110.14922	39.302357	1.4868	2.55E-09	3.09E-07	Homogentisate 1,2-dioxygenase
c52742_g1	0.4184647	107.30028	-8.0023	3.75E-24	1.39E-21	Polyphenol oxidase
c49788_g1	0.8369295	24.426437	-4.8672	4.19E-07	3.84E-05	Polyphenol oxidase
c52132_g1	269.44479	99.070106	1.4435	7.61E-20	2.15E-17	4-hydroxyphenylpyruvate dioxygenase
c93557_g1	175.6157	36.705672	2.2583	2.05E-23	7.23E-21	Aspartate aminotransferase
c39452_g1	343.55955	162.44681	1.0806	1.85E-16	4.20E-14	Phospho-2-dehydro-3-deoxyheptonate aldolase,
c53077_g2	13.065399	58.975541	-2.1744	2.35E-08	2.50E-06	Arogenate /prephenate dehydratase
c52184_g1	77.741448	28.123411	1.4669	7.18E-07	6.27E-05	phenylalanine ammonia-lyase 3
c52203_g1	16.785085	68.394023	-2.0267	9.02E-09	1.03E-06	Peroxidase
c48612_g1	1.9063393	56.862984	-4.8986	1.09E-14	2.15E-12	Peroxidase
c54004_g1	44.682734	14.215746	1.6522	4.23E-05	0.0026664	Peroxidase
c51559_g1	2.1853158	115.3544	-5.7221	7.00E-29	3.69E-26	Caffeic acid 3O methyl transferase
c44146_g1	72.301407	27.463237	1.3965	4.06E-06	0.0003196	caffeoylshikimate esterase
c41936_g1	726.31529	2840.6405	-1.9675	2.30E-289	4.27E-285	chalcone Synthase
c56396_g2	133.16478	47.488514	1.4876	5.60E-11	8.31E-09	ABF
c67578_g1	16.459613	119.27143	-2.8572	1.65E-20	4.91E-18	auxin-responsive protein IAA
c50421_g1	11.902997	45.99212	-1.9501	4.33E-06	0.000338	MYC2
c52417_g1	18.598433	56.334845	-1.5988	9.63E-06	0.0007055	Indole-3-acetic acid-amido synthetase GH3.5,
c1401_g2	391.35752	50.789384	2.9459	2.06E-66	3.71E-63	PP2C
c21743_g1	36.499424	2.7727307	3.7185	6.66E-09	7.74E-07	PP2C
c57241_g1	98.618189	30.059921	1.714	4.32E-10	5.80E-08	PP2C
c57287_g1	60.491402	3.3008698	4.1958	9.01E-15	1.79E-12	PP2C
c52897_g1	282.13822	56.598915	2.3176	1.04E-37	7.93E-35	PP2C
c53505_g1	340.63029	144.49008	1.2372	7.50E-20	2.12E-17	PP2C
c55258_g1	15.111226	141.62932	-3.2284	7.93E-27	3.65E-24	Pectinesterase
c54418_g1	48.309429	102.98714	-1.0921	8.72E-06	0.0006438	Pectinesterase
c48524_g1	13.716344	145.63438	-3.4084	9.01E-29	4.70E-26	Pectinesterase
c45767_g2	1.8598433	23.326147	-3.6487	4.77E-06	0.0003695	Pectinesterase
c67308_g1	11.345044	38.994276	-1.7812	6.98E-05	0.0041518	Pectinesterase
c46138_g1	12.135477	65.357222	-2.4291	3.23E-10	4.40E-08	Pectinesterase
c52600_g1	4.7426003	56.510891	-3.5748	1.60E-12	2.69E-10	Pectinesterase

**Fig 4:**
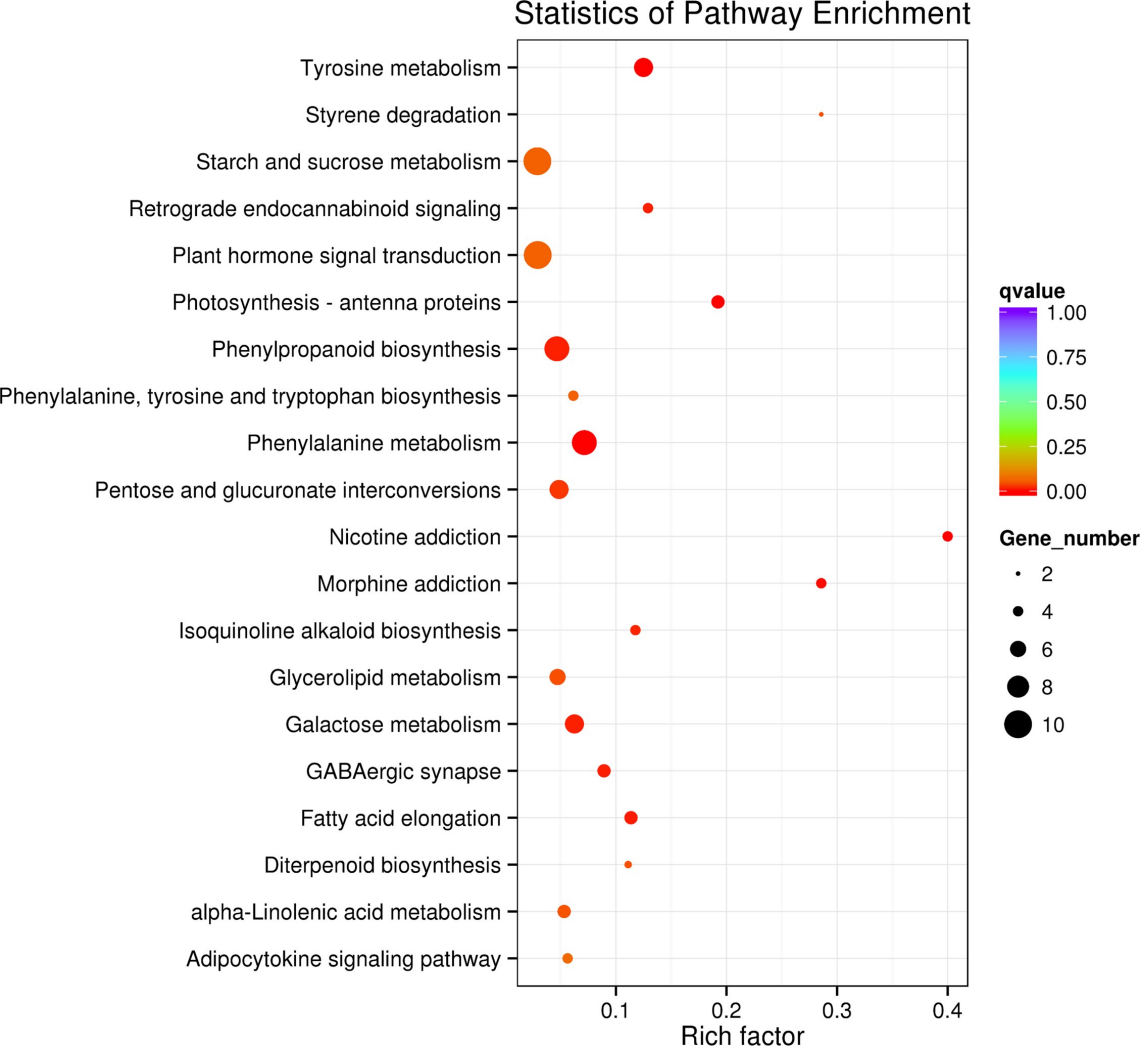
KEGG pathway of differentially expressed genes distinguished in Dis Vs Ck of gerbera. The pathway names are given in the vertical axis, rich factor in the horizontal axis, size of the point represents the number of DEGs and the shade of the dot represent the *q* value.

To understand the functional classification of DEGs, 14,335 unigenes belonging to 130 pathways were observed in Kyoto encyclopedia of genes and genomes (KEGG). KEGGs analysis indicated that maximum genes were differentially expressed for amino acid metabolism, carbohydrate metabolism, lipid metabolism, metabolism of cofactors and vitamins and metabolism of terpenoids and polyketides. These pathways are involved in tyrosine metabolism, tryptophan metabolism, phenylalanine metabolism, styrene degradation, phenylpropanoid biosynthesis, flavonoid biosynthesis and plant hormone signal transduction. Moreover, these pathways are interconnected with each other, genes involved in these pathways are highly enriched in diseased vs healthy plant samples and each was involved in different pathways in a different manner. The rich factor determins the ratio among the portion of pathway genes in the tested set and portion of pathway genes in the data set. It was observed that the *q-*value was higher for tyrosine metabolism followed by photosynthesis, phenylalanine metabolism, phenylpropanoid biosynthesis, and isoquinoline alkaloid biosynthesis (Fig 4). The top 17 KEGG pathways, and differently expressed genes involved in these pathways with least *p*-value are provoided in S3 Table.

### Candidate genes involved in phenylalanine, tyrosine and tryptophan biosynthesis in gerbera

**Fig 5:**
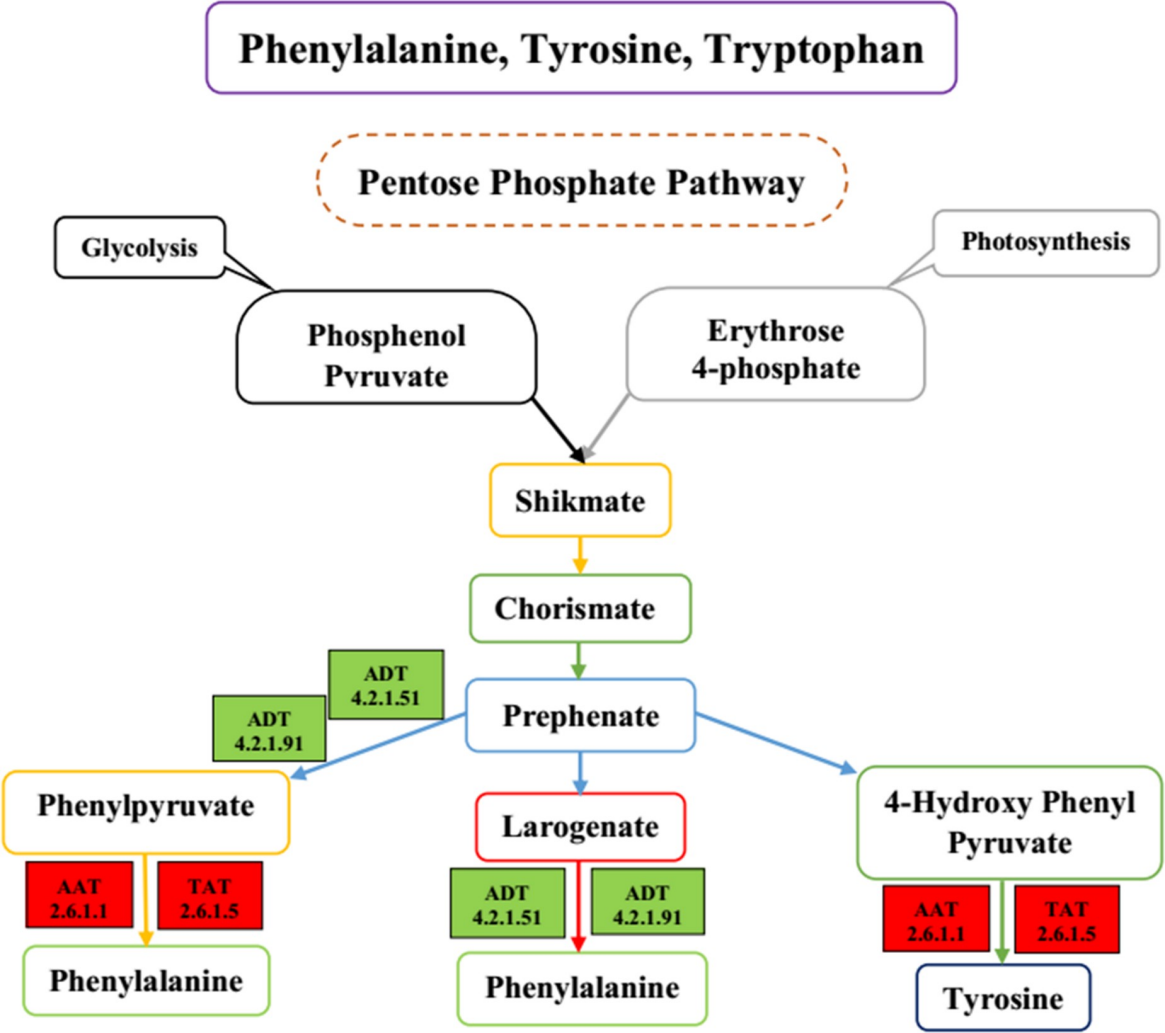
Schematic diagram of key genes and metabolites of phenylalanine, tyrosine and tryptophan biosynthesis in gerbera; Colored boxes represent the DEGs in this KEGG pathway. Red color represents the up-regulation of DEGs; Green color represents the down-regulation of DEGs.

Synthesis of aromatic amino acids, phenylalanine (Phe), tyrosine (Tyr) and tryptophan (Trp) starts with the alteration of phosphoenol pyruvate and erythrose 4-phosphate into chorismate by shikimate pathway. Four key enzymes involved in this pathway are 3-deoxy-D-arabino-heptulosonate-7-phosphate synthase (DAHP, EC: 2.5.1.54), aspartate aminotransferase (AAT, EC: 2.6.1.1), tyrosine aminotransferase (TAT, EC: 2.6.1.5), and arogenate dehydratase (ADT, E.C:4.21.91). Condensation of ethrose-4-phosphate with phosphoenolpyruvate is catalyzed through the enzyme DAHP. Gene annotated as *DAHP* was up-regulated. DAHP is a fundamental enzyme regulating flux through the shikimate pathway. Phenylalanine and tyrosine both are regulated from prephenate. Prephenate synthesizes the phenylalanine in two steps: one through phenylpyruvate as a metabolic intermediary and another is arogenate. Dehydration forming phenylpyruvate is catalyzed by ADT. DEG annotated as *ADT* was down-regulated with a log2fold change value of -2.20. DEGs involved in this pathway are presented in Fig 5. In Phe, Tyr, and Trp biosynthesis we also found genes that were annotated as *TAT* and *AAT*, these genes were up-regulated with a log2fold change value of 3.20 and 2.30, respectively in root rot-infected plants. DEGs related with this pathway, their identity, gene length, regulation, and *q*-value are presented in Table 4.

**Table 4:** KEGG annotation of enriched pathways in response to root-rot disease in gerbera.

KO ID	EC number	Gene-ID	Gene length	FC	q-value	Functional Annotation	Gene Regulation
**Tyrosine Metabolism**							
KI4454	2.6.1.1	c93557_g1	1661	2.2583	7.23E-21	Aspartate aminotransferase	Up-regulated
K00815	2.6.1.5	c54071_g1	1537	3.2292	1.22E-09	Tyrosine aminotransferase	Up-regulated
K00451	1.13.115	c55425_g1	1777	1.4868	3.09E-07	Homogentisate 1,2-dioxygenase	Up-regulated
K00422	1.10.3.1	c52742_g1	2096	-8.0023	1.39E-21	Polyphenol oxidase	Down-regulated
K01555	3.7.1.2	c53159_g1	1738	1.3843	1.15E-15	Fumarylacetoacetase	Up-regulated
K00457	1.13.11.27	c52132_g1	1717	1.4435	2.15E-17	4-hydroxyphenylpyruvate dioxygenase	Up-regulated
K00422	1.10.3.1	c49788_g1	2010	-4.8672	3.84E-05	Polyphenol oxidase	Down-regulated
**Phenylalanine, tyrosine and tryptophan biosynthesis**							
K01626	2.5.1.54	c39452_g1	1917	1.0806	4.20E-14	Phospho-2-dehydro-3-deoxyheptonate aldolase,	Up-regulated
K14454	2.6.1.1	c93557_g1	1661	2.2583	7.23E-21	Aspartate aminotransferase	Up-regulated
K00815	2.6.1.5	c54071_g1	1537	3.2292	1.22E-09	Tyrosine aminotransferase	Up-regulated
K05359	4.21.91	c53077_g2	1795	-2.1744	2.50E-06	Arogenate /prephenate dehydratase	Down-regulated
**Phenylpropanoid biosynthesis**							
K10775	4.3.1.24	c52184_g1	1085	1.4669	6.27E-05	Phenylalanine ammonia-lyase 3	Up-regulated
K10775	4.3.1.24	c52203_g1	1597	-2.0267	1.03E-06	Phenylalanine ammonia-lyase	Down-regulated
K01188	3.2.1.21	c54615_g3	1436	1.6777	2.95E-19	Hypothetical protein PHAV	Up-regulated
K00430	1.11.1.7	c48612_g1	1308	-4.8986	2.15E-12	peroxidase	Down-regulated
K00430	1.11.1.7	c17117_g2	1563	-3.4041	6.45E-205	peroxidase	Down-regulated
K00430	1.11.1.7	c57922_g3	1646	-3.4336	0.001789	peroxidase	Down-regulated
K00430	1.11.1.7	c54004_g1	1307	1.6522	0.002666	peroxidase	Up-regulated
K13066	2.1.1.68	c51559_g1	1344	-5.7221	3.69E-26	Caffeic acid 3O methyl transferase	Down-regulated
K18368	3.1.1.	c44146_g1	1517	1.3965	0.00032	Caffeoylshikimate esterase	Up-regulated
**Flavonoid biosynthesis**							
K00660	2.3.1.74	c41936_g1	1669	-1.9675	4.27E-285	Chalcone Synthase	Down-regulated
**Plant hormones and signal transduction**							
K14432		c56396_g2	2385	1.4876	8.31E-09	ABF	Up-regulated
K14484		c67578_g1	1122	-2.8572	4.91E-18	Auxin-responsive protein IAA	Down-regulated
K13422		c50421_g1	1777	-1.9501	0.000338	MYC2	Down-regulated
K14506		c52417_g1	1047	-1.5988	0.000705	Indole-3-acetic acid-amido synthetase GH3.5,	Down-regulated
K14497		c1401_g2	1748	2.9459	3.71E-63	Highly ABA induced PP2C gene	Up-regulated
K14497		c21743_g1	523	3.7185	7.74E-07	Protein phosphatase 2C	Up-regulated
K14497		c57241_g1	1773	1.714	5.80E-08	Protein phosphatase 2C	Up-regulated
K14497		c57287_g1	977	4.1958	1.79E-12	Protein phosphatase 2C	Up-regulated
K14497		c52897_g1	1506	2.3176	7.93E-35	Protein phosphatase 2C	Up-regulated
K14497		c53505_g1	1654	1.2372	2.12E-17	Protein phosphatase 2C	Up-regulated

### Transcripts related to tyrosine metabolism pathway in gerbera

**Fig 6:**
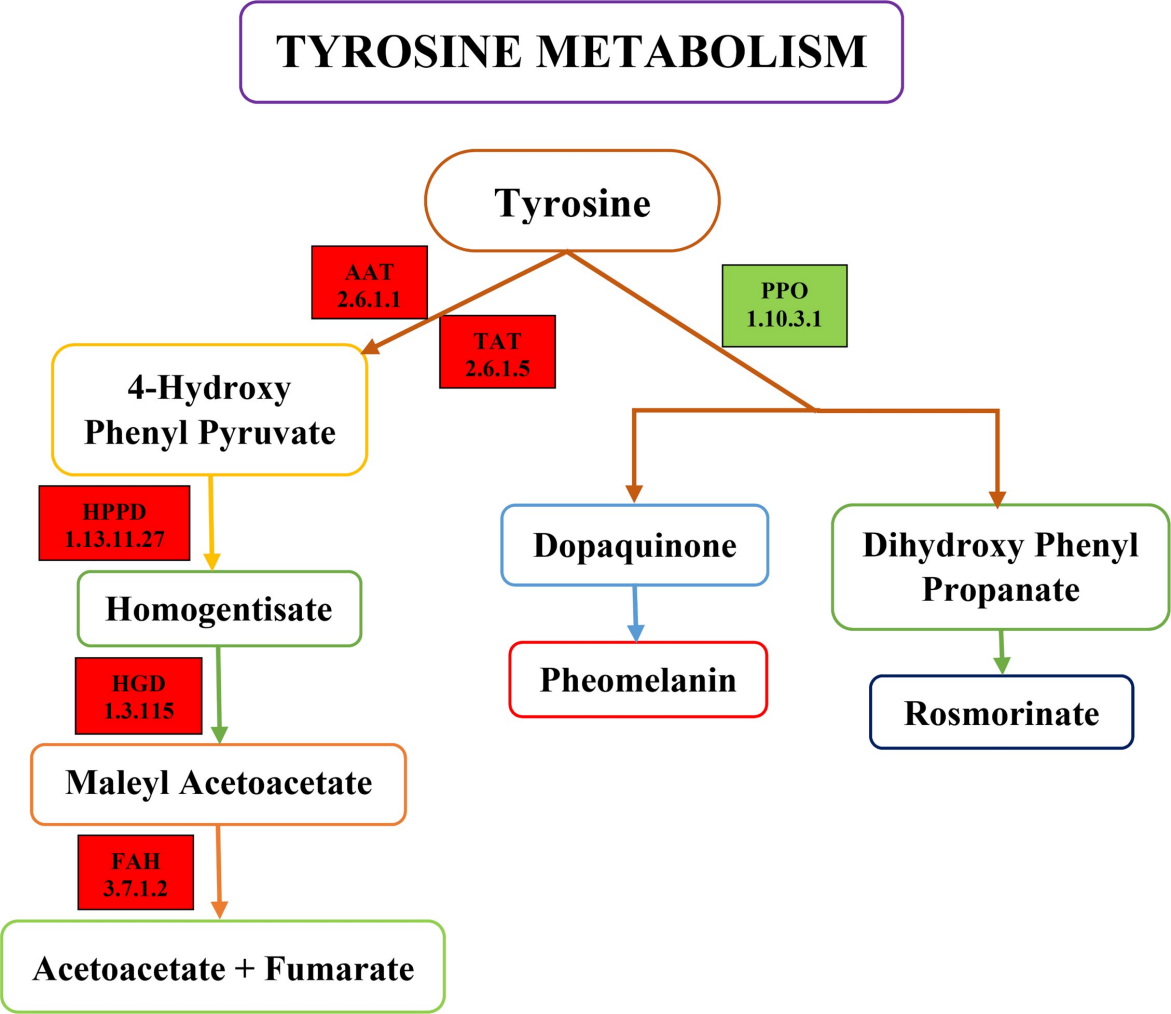
Schematic diagram of key genes and metabolites of tyrosine metabolism pathway in gerbera; Colored boxes represent the DEGs in this KEGG pathway. Red color represents the up-regulation of DEGs; Green color represents the down-regulation of DEGs.

Considering the EC of annotated sequences the enzymes associated with tyrosine metabolism were observed in response to disease infection. The pathways identified from KEGG incorporated all enzymes and metabolite, however, Tyr pathway was well characterized (Fig 6). Generally, six enzymes related with Tyr metabolism in plants are reported including Tyr aminotransferase (TAT, EC: 2.6.1.5), aspartate aminotransferase (AAT, EC: 2.6.1.1), 4-hydroxy-phenypyruvate dioxygenase (HPPD, EC: 1.13.11.27), homogentisate dioxygenase (HGD, EC: 1.13.115), fumeryl acetoacetate hydroxylase (FAH, EC: 3.7.1.2) and polyphenol oxidase (PPO, EC: 1.10.3.1).

Tyrosine aminotransferase and aspartate aminotransferase catalyze the conversion of Tyr into 4-hydroxyphenylpyruvate (4-OH-PhPyr). *TAT* and *AAT* genes were up-regulated with a log2fold change value of 3.20 and 2.20, respectively. 4-hydroxyphenylpyruvate then changed into homogentisate by enzyme HPPD (EC: 1.13.11.27). The gene annotated as *HPPD* was up-regulated with a log2fold change value of 1.40. Homogentisate acts as a center of the pathway, homogentisate is converted to 4-maleylacetoacetate by enzyme HGD (EC: 1.13.115) that is further converted to 4-fumarylacetoacetase by maleyl acetoacetate isomerase. *HGD* gene was up-regulated with a log2fold change of 1.50, and then 4-fumarylacetoacetate is hydrolyzed into two compounds acetoacetate and fumarate by enzyme FAH (EC: 3.7.1.2). These two compounds are the main product of amino acid metabolism. *FAH* gene was up-regulated with a log2fold change value of 1.40. Two *PPO* genes were down-regulated with log2fold change value of -8.00, -4.80 and q-value of 1.39e^-21^, 3.84e^-05^, respectively in response to root rot disease in gerbera. The genes in both pathways have very low (or zero) E-value. DEGs in this pathway, their description, gene length, regulation and *q*-value are shown in Table 4.

### Phenylpropanoid and flavonoid biosynthesis in gerbera

The five important enzymes in phenylpropanoid biosynthesis are phenylalanine ammonia-lyase (*PAL*, EC: 4.3.1.24), Beta-glucosidase (EC: 3.2.1.21), caffeic acid-3-methyl transferase (EC: 2.1.1.68), peroxidase (EC: 1.11.1.7) and caffeoylshikimate esterase (CSE, EC: 3.1.1). Phenylpropanoid pathway produces the compounds such as lignin and phytoalexins. PAL catalyzes the transformation of phenylalanine into cinnamic acid. In this pathway PAL, C4H and 4CL are indispensable enzymes, playing an important role in biosynthesis and affect the accumulation of phenylpropanoids in plants. *PAL* gene was up-regulated with a log2fold change value of 1.50 and another gene was down-regulated with a log2fold change value of 2.02. DEGs annotated as *Beta-glucosidase* and *caffeoyl-o-shikimate* and *peroxidase* were up-regulated with log2fold change values of 1.70, 1.40 and 1.70, respectively. *Caffeic acid-3-methyl transferase* was down-regulated with a log2fold change value of -5.70. Similarly, three *peroxidase* genes were also down-regulated with log2fold change values of -4.90, -3.50 and -3.50, respectively in response to rootrot disease infection.

**Fig 7:**
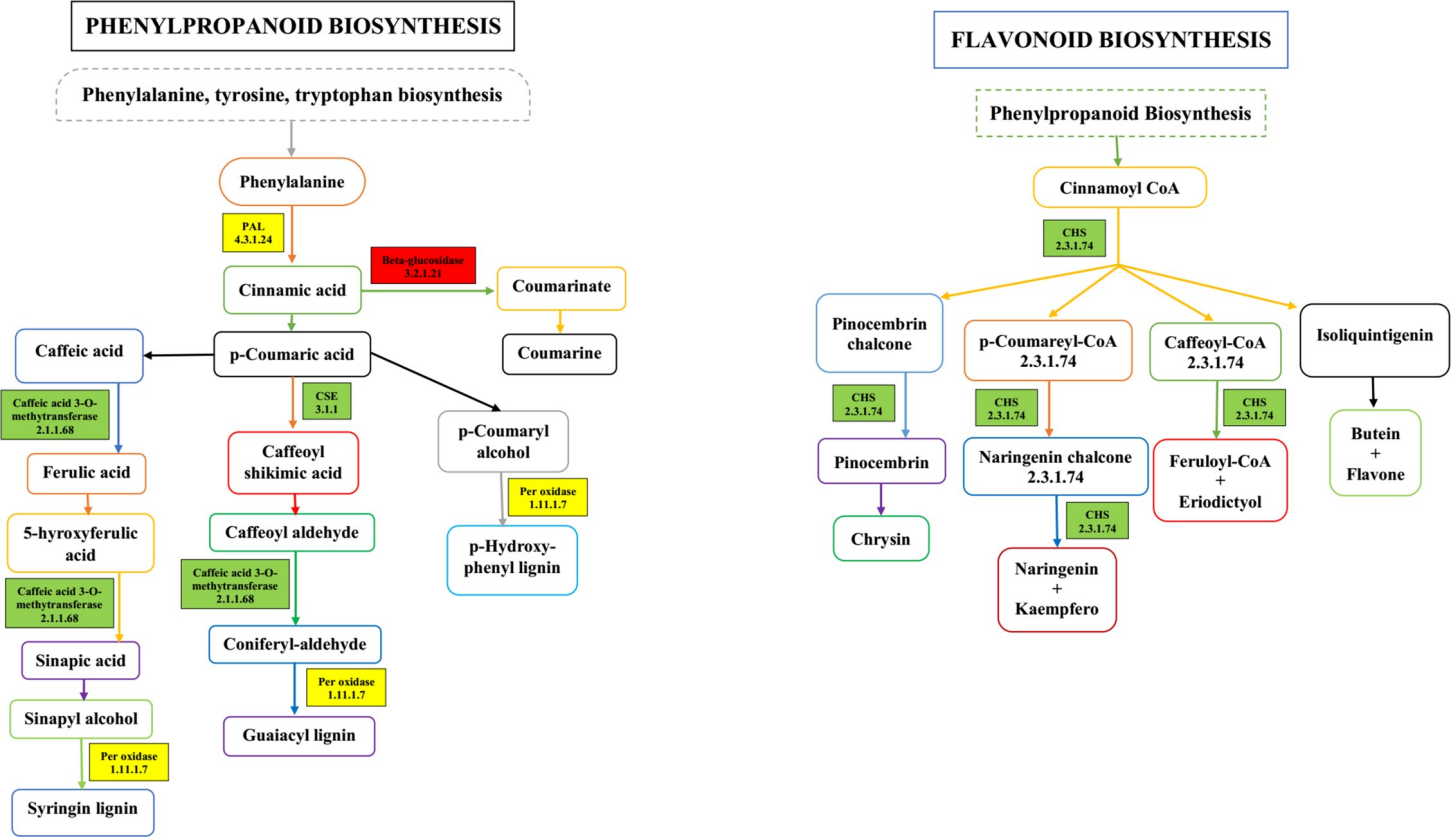
Schematic diagram of key genes and metabolites of phenylpropanoid biosynthesis & flavonoid biosynthesis pathway; Colored boxes represent the DEGs in KEGG pathways. Red color represents the up-regulation of DEGs; Green color represents the down-regulation of DEGs; Yellow color represents the up-and down regulation of DEGs.

Flavonoid biosynthesis includes iso-flavonoid biosynthesis, anthocyanin biosynthesis, and flavone and flavonol biosynthesis. Flavonoids are generated from phenylpropanoid pathway that converts phenylalanine into 4-coumaroyl-CoA which is primary branch point in phenylpropanoid pathway. Either it is utilized in flavonoids biosynthesis (CHS catalyze the flavonoid skeleton and leads to the synthesis of flavonol, cyaniding, and anthocyanin) or produces methoxylated guaiacy (G), syringyl (S) monolignols and lignins. The first enzyme for this pathway is chalcone synthase (CHS, EC: 2.3.1.74) that makes chalcone scaffolds and all flavonoids are derived from it. In flavonoid biosynthesis, one DEG annotated as *chalcone synthase* was down-regulated with a log2fold change value of 2.00, in response to disease infection (Fig 7). The genes in both pathways have very low (or zero) E-value. Genes in these pathways, their description and their expression are shown in Table 4.

### Plant hormones and signal transduction in gerbera

**Fig 8:**
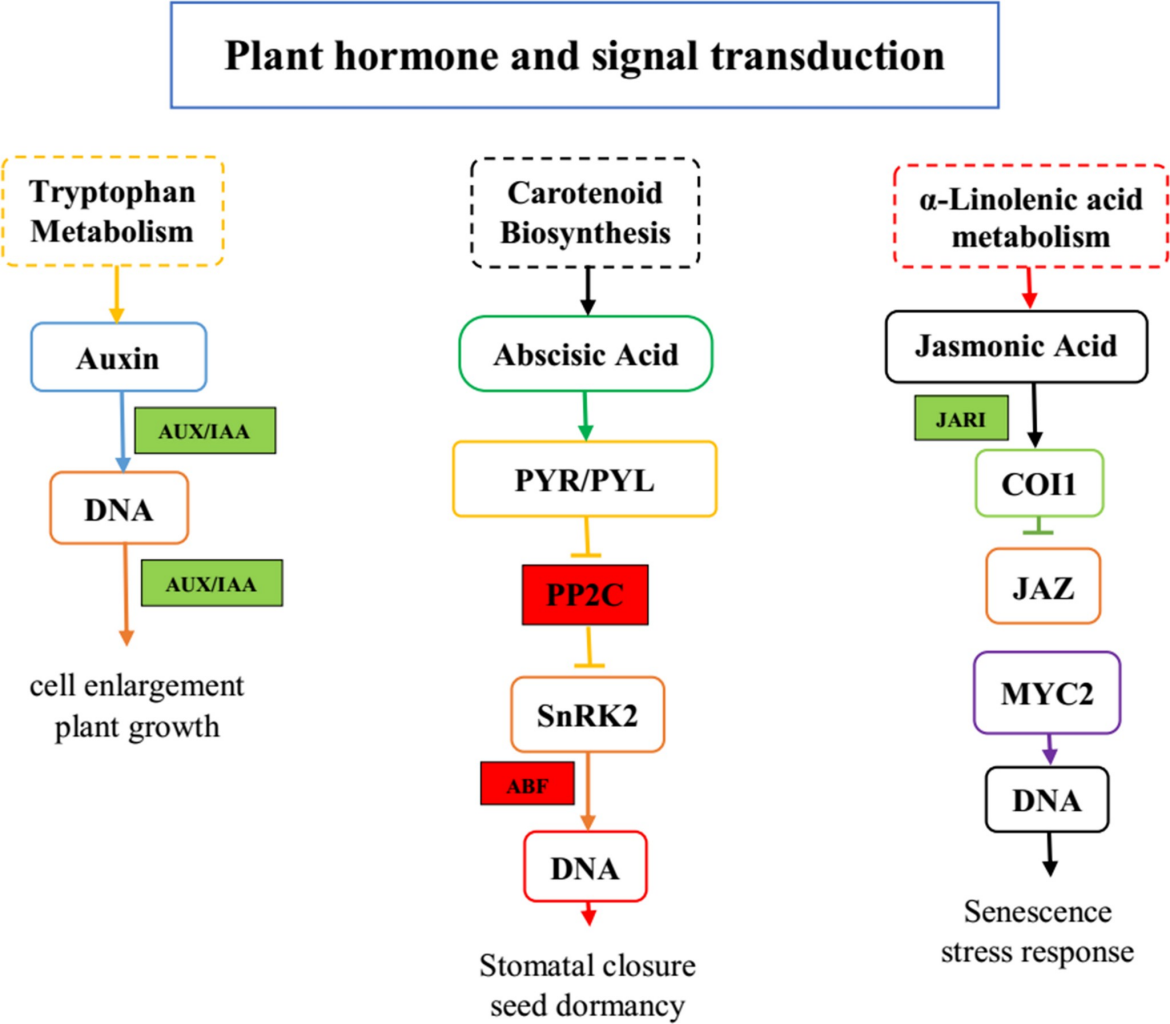
Schematic diagram of key genes and metabolites of plant hormones and signal transduction pathway in gerbera upon root-rot infection. Colored boxes represent the DEGs in KEGG pathway. Red color repreents up-regulation of DEGs; Green color representsdown-regulation of DEGs.

Plants produce a lot of hormones such as auxins, cytokinins (CK), gibberellins, abscisic acid (ABA), ethylene (ET), salicylic acid (SA), jasmonate (JA), and brassinosteriodes (BR). The five key regulatory enzymes in this pathway were ABF (c56396_g2); IAA (c67578_g1); MYC2 (c50421_g1); JAR1 (c52417_g1); PP2C (c1401_g2, c21743_g1, c57241_g1, c57287_g1, c52897_g1, c53505_g1) (Fig 8). Gerbera transcriptome data contains multiple contigs encoding these enzymes. These genes are involved in senescence, the stress responses in gerbera plant is related to jasmonic acid and α-linolenic acid metabolism. DEGs in plant hormone signal transduction pathway annotated as *JAR1*, *MYC2*, and *IAA* were down-regulated with log2fold change values of -1.60, -2.00 and -2.90, respectively. Six DEGs, annotated as *PP2Cs*, were up-regulated with log2fold change value of 3.00, 3.70, 1.70, 4.20, 2.30 and 1.00 in response to root rot disease (Table 4).

### Validation of the sequencing data by qRT-PCR

**Fig 9:**
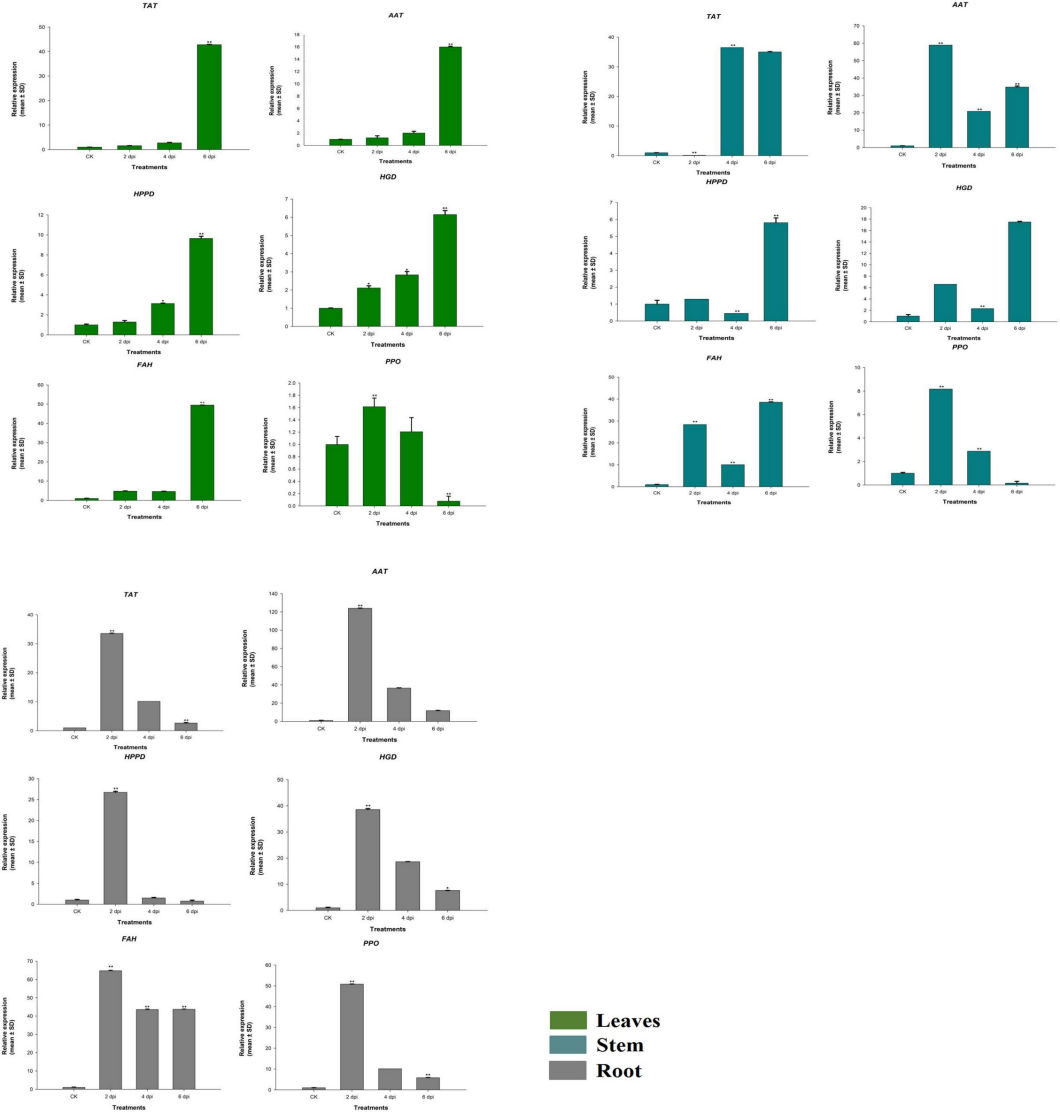
Expression analysis of various genes in tyrosine metabolism pathway in combination of root-rot pathogen inoculation and without inoculation in leaf, petiole and root tissues of gerbera plant. Control (C) untreated; two, four, six days of post-inoculation of the pathogen in leaf, petiole and root tissues in *GhTAT*, *GhAAT*, *GhHPPD*, *GhHGD*, *GhFAH* and *GhPPO* genes. Relative expression was investigated using the mean of CT values that were standardized to the mean CT estimation of the housekeeping gene 18S. The expression standards were determined using equation 2^-ΔΔct.^ Data are presented as Mean _±_ SD of autonomous biological replicates (n = 3 biological replicates) (LSD test *p* < 0.05). Asterisks show substantial changes between non-pathogenic and pathogen-inoculated means within the identical time of specimen, as per one-way ANOVA (** represents significant variance at *p* < 0.01 and * represents significant variance at *p* < 0.05).

To indorse the RNA-Seq expression profiles of gerbera DEGs obtained by RNA-Seq, the expression pattern of six identified responsive genes was estimated by qRT-PCR utilizing gene-specific primers. The expression comparisons were performed between healthy (leaf, petiole and root) plant samples and *phytophothora cryptogea* inoculated (leaf, petiole and root) plant samples after two, four and six days of inoculation. The results showed that the genes were significantly affected by disease infection by different days after pathogen inoculation compared with control. We compared the expression of genes from tyrosine metabolism pathway that are *GhTAT*, *GhAAT*, *GhHPPD*, *GhHGD*, *GhFAH* and *GhPPO* after 2, 4 and 6 days of inoculation with *phytophothora cryptogea* in leaf, petiole and root tissues. *GhTAT*, *GhAAT*, *GhHPPD*, *GhHGD*, and *GhFAH* gene expression was relatively higher after 6 dpi in leaf tissues. The expression was highly significant (*p* < 0.01) in all genes compared with control. *GhHPPD*, *GhHGD* and *GhFAH* gene expression were higher at 6 dpi in the leaf and petiole tissues. In petiole tissues, *GhHPPD*, *GhHGD*, and *GhFAH* showed higher relative expression after six dpi. The expression of *GhHPPD*, and *GhFAH* was highly significant (*p* < 0.01) at 6 dpi compared with control. While G*hTAT* and *GhAAT* gene expression were highly significant at 4 dpi and 2 dpi, respectively. We observed that for most of the genes the expression was increased with the time of infection. In root tissues, *GhTAT*, *GhAAT*, *GhHPPD*, *GhHGD* and *GhFAH* gene expression was high after 2 dpi. *GhPPO* gene expression was high at 2 dpi in (leaf, petiole and root) tissues and continuously decreased later on. The qRT-PCR expression summaries of these six gerbera genes was similar with RNA-seq information. Overall, the enrichment in expression level and defense reaction revealed by RNA-Seq results in response to be pathogenic contamination were similar with qRT-PCR, affirming the consistency of sequencing data and our results (Fig 9).

## Discussion

In order to improve the understanding of transcriptomic responses of healthy and diseased gerbera plants to *Phytophothora cryptogea*, we performed RNA-Seq investigation to look at the genome-wide expression pattern of gerbera plants with pathogenic and non-pathogenic separates. Our study provides the first prominent evidence regarding the gene expression pattern when gerbera plants are infected with *Phytophothora cryptogea* (root rot causing agent).

In this investigation, we established transcriptome response (leaf and root tissues) of *Gerbera hybrida* cv. Daxueju for healthy and diseased plants. A total of about 114 million reads were generated from healthy (59 million) and diseased (55 million) plant samples respectively, constituting approximately 8.55 Gb and 8 Gb of cDNA sequence data. After *de novo* association, we produced 141,972 transcripts, with an average size of 727 bp and N50 length of 1,274. The highest numbers of homologs were found for *Vitis vinifera* (grapevine). Remarkably, the grapevine is a alos known to produce high amount of secondary metabolites and its association with *p*. *cryptogea*. Numerous studies have been performed on this crop-pathogen from different perspectives [13] and [1] that could be valuable for the interface of pathogen with gerbera also.

### Plant metabolism and pathogen interaction

Plant metabolism and pathogen contamination are firmly interconnected. Pathogens require nourishment from the host for colonization and in this manner accessibility of nutrition can be influenced by plant metabolism [14, 15]. Plant shield responses to pathogen that require energy which is primarily resulted from primary metabolic process. Principal metabolites also work as signaling molecules to trigger the resistance reactions following pathogen acknowledgment and signal transduction metabolites [16]. Nitrogen impacts the result of host-pathogen association. Plant nitrogen is generally put away in N-transport amino acids, for example, asparagine, aspirate, and glutamate. These plant-produced amino acids are significant N basis for fungal growth and development. The pathogen may even influence plant metabolism to facilitate them to get nitrogen-rich mixes [17]. It was suggested that high N accessibility could encourage construction of constitutive and induced resistance molecules [18]. Several genes involved in N-assimilation were differentially up-regulated in tomato [19] and tobacco [20].

It is considered that some natural secondary metabolites play an important role in plant defense mechanism [14, 21]. Part of amino acid metabolism, responsible for plant–pathogen association is emphasized by current studies that exploits that mutants influence enzymes and carriers of amino acid metabolism. Amino acids are tangled in cellular responses and influence physiological processes such as plant growth, intra-cellular pH, metabolism energy, and resistance against biotic and abiotic stresses [22, 23, 14, 24]. In different plant parts, function and degradation of amino acids [25, 26] and signaling in plants has been debated [27, 28]. Some amino acids (Phe, Trp and Tyr) assist as an originator for the production of secondary metabolites, long-chain aromatic acids, such as methionine, alanine and other aromatic acids [29]. We have identified 30 DEGs involved in KEGG pathways, most of these are associated with the metabolism. These were also differentially expressed in diseased plants compared with control and encoded for those enzymes that are important for defense-related mechanisms. We focused on Tyr metabolism pathway. The sequences similarities of the best hits for those transcripts are above 85% and best hits come from other species within Asteraceae such as *Halianthus annu*s, *Lactuca sativa*, and *Cynara cardunculus* indicating that these pathways are well-preserved within this family.

### Metabolism of tyrosine in plants

**Fig 10:**
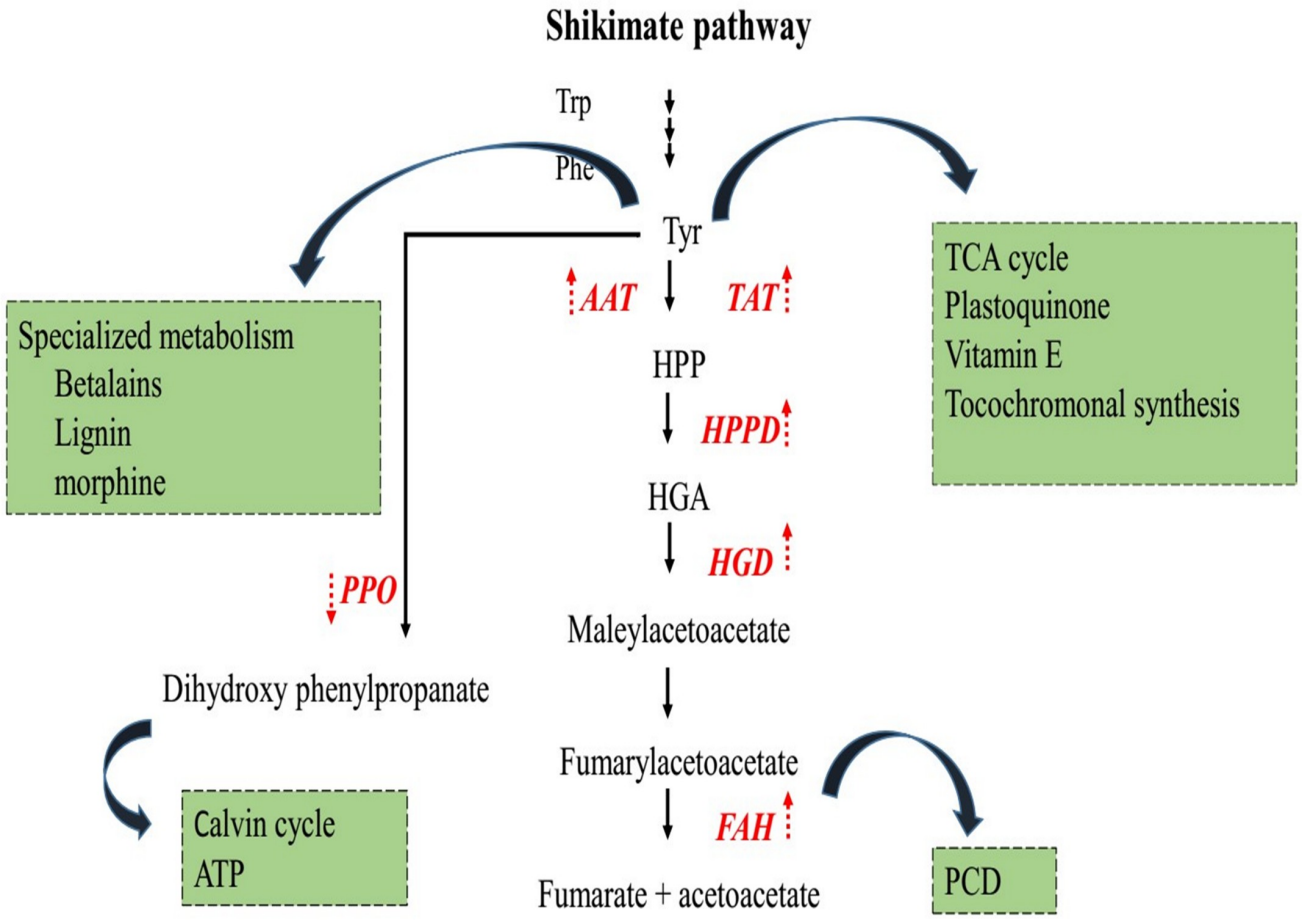
Biosynthesis and metabolism of tyrosine in plants. Shikimate pathway gives the originator for Tyr as well as other AAA; trp and phe. Tyr can be assimilated into a protein that catabolized into TCA cycle; metabolized into vitamin E and plastoquinone; or various plant natural products. Synthesis of tyrosine shown by black arrows. Genes are shown in red color; regulation of genes in tyrosine metabolism are shown with red arrows. Abbreviations Trp, tryptophan; Phe, phenylalanine; Tyr, tyrosine; HPP, hydroxyphenylpyruvate; HGA, Homogentisate; TAT, tyrosine aminotransferase; AAT, aspartate aminotransferase; HPPD hydroxyphenylpyruvate dioxygenase; HGD, homogentisate dioxygenase; FAH, fumaryl acetoacetate hydroxylase; PPO, polyphenol oxidase.

In plants, biotic and abiotic stresses lead to the production of reactive oxygen species (ROS). Fortunately, plants possess different enzymatic and non-enzymatic oxidative defense system that helps protect plants by scavenging ROS. Tyr is an aromatic amino acid that is a prerequisite for protein synthesis in almost all organisms particularly plants and microorganisms. It is the originator of several secondary metabolites such as alkaloids, phenylquinones, cyanogenic glycosides and other amino acids such as methionine that have different physiological roles as antioxidants, attractants, electron carriers, and defense compounds [30]. Tyr catalyze the reversible transmission of Tyr and HPP via PLP cofactor [31]. This is primary step of Tyr metabolism to yield plastoquinone and tocopherols and considered as essential enzyme in carbohydrate metabolism, biosynthesis, degradation of amino acids and several other metabolic pathways [29, 32] that feed into the tricarboxylic acid (TCA) cycle. Therefore, it is considered a fundamental candidate among disease-resistance related genes. The inimitable variation of Tyr biosynthetic pathways in various plants likely help the construction of downstream specialized metabolites that assist plants to adjust and survive under different environmental conditions. Tyr is a center point to a myriad of specified metabolic pathways that are facilitated by various Tyr-utilization enzymes. These enzymes include tyrosine aminotransferase (TAT, EC 2.6.1.5) and aspartate aminotransferase (AAT, EC 2.6.1.1), 4-hydroxyphenylpyruvate dioxygenase (HPPD, EC 1.13.11.27), homogentisate 1, 2 dioxygenases (HGD, EC 1.13.115), fumarylacetoacetase (FAH, EC 3.7.1.2), and polyphenol oxidase (PPO, EC 1.10.3.1) (Fig 10).

### Tyrosine aminotransferase (Tyr-AT) activity in plant defense

The activity of Tyr-AT has been identified in many plant species comprising *Psium sativum* and *Anchusa officinalis*. Genes encoding Tyr-AT enzymes have been identified recognized for numerous plants [33]. Recombinant AAA aminotransferase enzymes from opium poppy (*Papaver somniferum*) and melon (*Cucumis melo*) are able to catalyze the transamination of Tyr to HPP [34]. An AAA aminotransferase from *Arabidopsis* and *Petunia hybrid* support Tyr transamination to HPP over the contrasting response [35]. Tyr and Phe pathways are biochemically joined by these Tyr ATs. Consistence with *Arabidopsis thaliana TAT1* specially catalyzing Tyr transamination to HPP, *Arabidopsis tat1* mutants have expanded Tyr and reduced tocopherol levels [36] demonstrating that *AtTAT1* is involved in Tyr metabolism. RNAi suppression of *Opium poppy* Ps TyrAT decreased downstream BIAs [34]. TAT activity was found to be activated by coronatine, octadecanoids methyl jasmonate (MeJA), 12-oxophytodienoic acid (MeOPDA), herbicide oxyfluorfen, wounding, UV light and high light [37, 38]. The full length cDNAs microarray analysis of Arabidopsis genes showed that tyrosine aminotransferase responsive genes were up-regulated in high salinity, cold and drought stress [39]. We found several *TAT* genes; among them one DEG (c54071_g1) in tyrosine metabolism pathway was obviously up-regulated after pathogen infection. The expression of *GhTAT* gene was increased in leaves and petioles with the infection time.

### AAT involved in Amino acid metabolism during infection

Aspartate aminotransferase is involved in plant carbon and nitrogen dissemination that catalyzes the reversible response of transmembrane among aspartate and 2-oxoglutarate to yield oxaloacetate and glutamate. AAT's contain additional prephanate dehydratase/prephenate dehydrogenase domains in N-ends that are exceptional to oomycetes. *AAT* gene is engaged with pathogen pathogenicity and nitrogen utilization during contamination. Asparagine levels were associated with early defense responses. However, alteration of aspartate to asparagine can modulate plant defense [40]. AAT enzyme activity has been recognized in higher plants, fungus, animals, bacteria but less studied in oomycete. It was also identified in *Phytophthora infestans* and *P*. *sojae*. Two novel motifs related to prephenate dehydratase and prephenate dehydrogenase were identified, that were also related to tyrosine and phenylalanine metabolism [41]. Aminotransferase is associated with defense mechanisms. The improved resistance of wild-type melon PI against *Pseudoperonospora cubensis* was combined with improved glyoxylate aminotransferase activity: it was proposed that the upstream photorespiratory production of glyoxylate from glycolate, discharging H_2_0_2_, assumes a central role in hindering pathogen [42].

Gene expression analysis revealed that the aspartate aminotransferase encoding genes were differentially expressed when exposed to biotic stress such as infection with *Phytophthora infestans*, *Botrytis cinerea*, *Pseudomonas syringae* and abiotic strains [43]. In tomato, Asparagine synthase showed a significant role in immune mechanism, in response to *B*. *cinerea* infection. *AAT* have been overexpressed in response to *B*. *cinerea infection* in Arabidopsis [44]. Micro-array exploration of infected and non-infected strains and wild type tomato plants demonstrate that *AAT* was up-regulated by disease infection [45]. In soybean plant, the exposure of hyphae to soybean leaves after 1.5, 3, 6, 12 and 24 hpi were analyzed. *AAT* gene showed high expression during inductions, and the maximum expression level was observed after 24 hpi. *AAT* gene was supposed to play vital role in pathogenicity because it displaced plant-induced expression pattern [46]. We found *AAT* gene (c93557_g1) was up-regulated in response to root-rot infection proposing that biosynthesis of these amino acids is mandatory by pathogens during the infection. *GhAAT* expression was continuously increased in leaves with infection time demonstrating a credible role in plant defense mechanism.

### Vitamin E (tocopherols and tocotrienol derivatives) involved in plant defense

4-hydroxyphenylpyruvate dioxygenase and homogentisate are involved in biosynthetic pathway of tocopherol in plants that leads to the production of plastoquinone and vitamin E. In plants, the product of HPPD response is a key forerunner for biosynthesis of photosynthetic pigments such as plastoquinones and vitamin E (tocotrienol and tocopherols products) [47]. The generic term vitamin E is used for large number of effective lipid-soluble antioxidants. These antioxidants are tocochromanols; tocopherols, tocotrienols, and plastochromanols-8 [47]. Tocopherols have an important role in various physiological processes under stress conditions and involved in ROS-scavenging alongside different antioxidants such as glutathione and ascorbate [48, 49]. Construction of homogentisate-resultant metabolites are indispensable for the assurance of plant cells against oxidative destruction during biotic and abiotic stress condition, photosynthesis, seed desiccation and storage [26]. Homogentisate is derived from chorismate pathway and these are the final product of shikimate pathway [50, 51]. Genes encoding *HPPD* have been distinguished from various plant species comprising *Dacus carota*, *Arabidopsis thaliana* and *Hordeum vulgare*. In Arabidopsis, the levels of α-tocopherol and γ-tocopherol increased by 3.60 and 13.5 fold under dehydration situation [52]. Upon the attack of bacterial pathogen *Pectobacterium chrysanthemi*, the expression of *HPPD* gene was increased abruptly in sweet potato leaves [53]. Transgenic plant intended to upstream homogentisate amassing by collaborating microbial enzymes that bypass this reaction inhibition leading to increased vitamin E production. Seed explicit expression of *E*.*coli* TyrA, *HPPD* resulted in increase of homogentisate and tocochromonal levels in Arabidopsis seeds [54]. These biofortification endeavors determined that the key factor impending vitamin E biosynthesis in plants is the availability of homogentisate. In tobacco plant, the rise in vitamin E content was detected due to overexpression of barley *4-hydroxyphenylpyruvate dioxygenase* gene [55]. In tobacco and soybean plants *HPPD* gene was overexpressed from *P*. *fluorescens* and this provided the high resistance from herbicide [56]. *HPPD* gene was significantly enriched in mature leaves of *Lucta sativa* under stress conditions. The mRNA expression level was upregulated by 12-fold and 4-fold enrichment was observed for α-tocopherol content compared with control [57]. Categorization and expression reporting of *4-hppd* from *Salvia miltiorrhiza* hairy root cultures indicated that it constitutively expressed in stems, leaves and roots. Expression level under different stress conditions indicates that *Smhppd* expressed constitutively in all tissues with maximum expression in roots and minimum in the stem. MeJA and SA likewise boost *Smhppd* expression level that was greater on 6^th^ day after treatment compared with control. The expression of *hppd* gene in *Salvia miltiorrhiza* was down-regulated in response to Ag+ while yeast extract up-regulated *hppd* transcript level in the course of culture period [58]. We found that the *HPPD* and *HGD* gene (c52132_g1; c55425_g1) in tyrosine metabolism pathway were up-regulated after infection. qPCR expression analysis of *GhHPPD* and *HGD* gene showed that in leaf and petiole tissues the expression was increased with the infection time.

### Loss of FAH in tyrosine metabolism causes cell death in plants

Fumaryl acetoacetate hydrolase (FAH) hydrolyzes fumaryl acetoacetate to fumarate and acetoacetate that is the last step in tyrosine degradation pathway and is necessary in animals and plants metabolism [59], [33]. Deficiency of FAH causes programmed cell death (PCD) in plants that goes to the removal of particular cell, tissues, or whole organ. In plants, PCD had a vital role in biotic stress response, immunity, senescence, and defense as well as in developmental processes. Hypersensitive response is one of the well-characterized examples of PCD that occurs during the incompatible plant-pathogen interaction and hinders the spread of pathogen zoospores [60, 61]. In *Arabidopsis thaliana*, loss of FAH in tyrosine metabolic pathway leads to impulsive cell death under short day circumstances. According to a report, expression of Tyr degradation pathway genes was up-regulated in *sscd* (short day sensitive cell death) mutant [62]. In our study, DEG annotated as *FAH* (c53159_g1) in tyrosine metabolism pathway was up-regulated in RNA-Seq and qRT-PCR data. The expression was high at later stages of infection compared with initial stages. This suggests that the root rot infection enhances the tyrosine degradation activity that helps gerbera plant resist against infection.

### PPO activity in defense mechanism

Polyphenol oxidase is an oxidoreductase that catalyzes the oxidation of moniphenols, it assumes a crucial role in the biosynthesis of secondary metabolites such as aurones and betalins. PPO has a precise role in plant protection against insects and pathogens. It performs different functions in plants. In transgenic tomato plants, *PPO* overexpression greatly increases resistance to *Peudomonas syringae* [63] and the manipulation of *PPO* activity provides resistance against diseases and insect pests. A recent study illustrated that *PPO* overexpression delays fungal infection. Its expression and transcript level were persuaded by mechanical injury, pathogen infection, fungal and bacterial pathogenesis, and JA or salicylic acid in plant defense mechanisms [64, 65]. The silence of *PPO* gene enhanced the plant photosynthesis by stimulating the glycolysis process, regulating Calvin cycle and giving ATP for energy metabolism [66]. *PPO* activity *in vivo* typically occurs in damaged plant tissues that have lost cellular compartmentalization. The expression and activity of *PPO* increases during tissue browning in apple and litchi [67, 68] and reducing the expression of *PPO* reduces the browning rate in transgenic potatoes.

PPO is a significant supporter in plant fundamental resistance against pathogens that contributes to catalyzing phenolic oxidation to restrict disease movement. Subsequently it can be associated with systemic resistance stimulation against plant infection [69]. *PPO* injected with *M*.*oryzae* represented elevated amounts of PPO activities in contrast with non-inoculated plants [70]. According to a report, wounding and herbivore attacks promote PPO activity [71]. In tomato plants, induction of antisense PPO cDNA induces resistance in plants to pathogen *Pseudomonas syringae* that results in down-regulation of *ppo* gene family members [72]. The expression of *PPO* gene was considerably decreased in response to wounding, MeJ and herbivory in transgenic tobacco [73]. Our study revealed the presence of two *PPO* DEGs (c49788_g1; c52742_g1) in Tyr metabolism pathway were down-regulated. The expression of *PPO* gene in leaves, petioles and roots was high at 2 dpi while it decreases later.

The expression of *GhTAT*, *GhAAT*, *GhHPPD*, *GhFAH* in root was different. The expression was high at an early stage and decreased at later stages that is similar with a previous study in which root expression of defense-related genes was observed in wheat in response to *F*. *culmorum* infection. The *F*. *culmorum* infection was greater at 24 hpi but somewhat low at 48 and 96 hpi [74]. The differential expression of all these genes showed that these played a vital part in plant resistance mechanism.

## Material and methods

The *Gerbera hybrida* cv. Daxueju seedlings grown in Sanming Modern Agriculture Sci-tech Demonstration Garden, Sanming, Fujian was utilized as plant material for transcriptome analyses. In this study, the samples of healthy and root-rot infected plants were harvested for transcriptome analyses. Leaves and roots of healthy and diseased plants were collected in three biological replicates. Samples were prepared as healthy gerbera plantlets and diseased gerbera plantlets and Illumina sequencing were performed for both samples.

## Transcriptome analysis

### RNA isolation and cDNA library preparation

**Fig 11:**
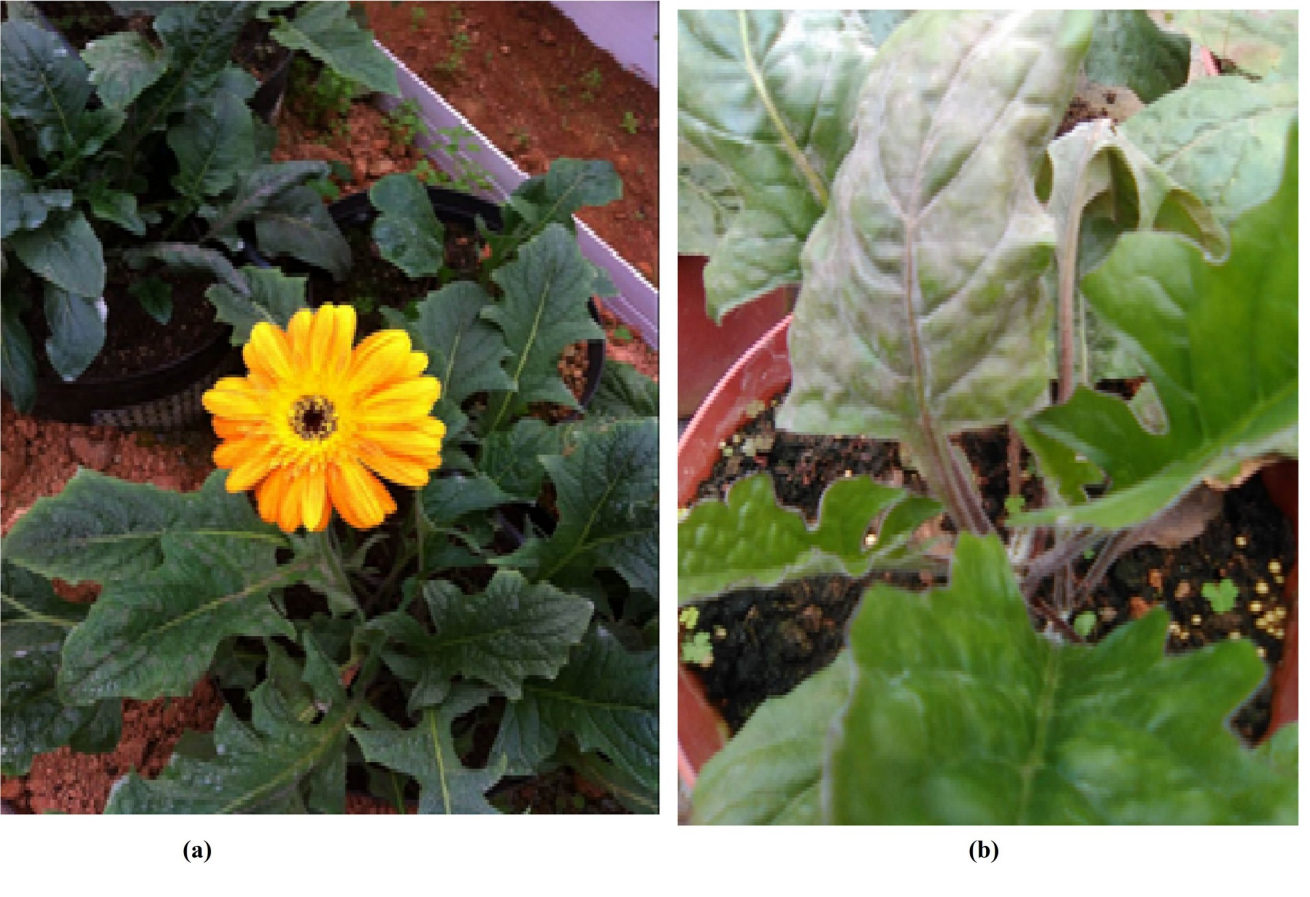
(a) Healthy and (b) root-rot diseased gerbera plant.

The *Gerbera hybrida* cv. Daxueju seedlings grown in Sanming Modern Agriculture Sci-tech Demonstration Garden, Sanming, Fujian were used for cDNA sequencing. Healthy and root rot diseased plant samples were gathered. The leaves and roots of healthy and infected plants were taken and temporarily stored at -80°C prior to RNA-Seq analysis (Fig 11). Frozen tissues were crushed in liquid nitrogen with mortar and pestle. Total RNA of the samples was extracted using Trizol reagent and afterward purified with the RNA cleanup procedure according to manufacturer’s instruction. Total RNA of the leaves and roots (three replicates) was mixed in equivalent quantities. RNA quality and quantity were determined using a Nanodrop 2000 and checked by RNase free agarose gel electrophoresis. Additionally, the total RNA of the samples were evaluated for quality using an Alignment 2100 Bioanalyzer TM. RNA-Seq libraries were prepared by Illumina HiSeq technology.

### Sequencing, assembly and annotation of the transcriptome

The cDNA libraries of both samples (healthy and root-rot diseased plant) were sequenced by 2×100 bp paired-end sequencing on an Illumine HiSeq platform according to manufactures instructions. The primary base setup and quality separation of the image statistics were refined by means of default parameters in the Illumine data processing pipeline. The adaptors, any anonymous nucleotides greater than 10% and low quality reads containing more than 50% bases with Q-values ≤ 20 were executed after sequencing. Processed reads with an identity of 95% and coverage length of 200 bp were accumulated using Trinity software [75], that comprises three modules that are Inchworm, Chrysalis and Butterfly. Initially, the software consolidates reads of specific lengths of overlap to make longer fragments termed contigs. At that point, the reads were mapped back to the contigs that were associated up to extended on neither end. The acquired sequences were characterized as unigenes subsequent to eliminate any redundancy. The redundant sequences were set up as the identity of overlapping parts which was reached at 94% with the lengths prominent than 200 bp.

The unigenes were submitted to protein databases for annotation and homolog correlation by BLASTX algorithm (e-value ≤1e-5) comprising Nr, Nt, Swiss-prot, GO and KEGG database. The Blast2GO v 2.5 [76] and HMMER v 3.0 software were utilized for Gene ontology (GO) annotation and functional sorting. BLASTN was used in the Nt nucleotide database. GO annotation in Blast2GO comprised of three phases, blasting, mapping and annotation. The assembled contigs were matched by BLASTX in contrast to the NCBI non-redundant protein database (nr) using Blast2GO. The expectation value (E-value) threshold was set at 1e^-5^ for nr, nt and KOG while le^-10^ for KEGG. Subsequent to blasting, Gene Ontology (GO) terms related to the hits were recorded. At the point when a Blast result is effectively mapped to single or numerous GO terms. GO annotations were allotted. Enzyme code (EC) annotation was accessible only for contig sequences with GO annotation. Also, the KEGG mapping was done to show enzymatic roles in the context of metabolic pathways in which these take an interest.

### GO and KEGG development study of differentially expressed genes

The GO enhancement investigation of differentially expressed genes was prepared by GOseq R package, in which the gene length was balanced. GO terms with adjusted *p*-value < 0.05 were considered differentially expressed genes. KEGG is a database for understanding high-level utilities and elements of the biological system such as cell, organism, and ecosystem from molecular dimension data, mainly large-scale datasets are produced by genome sequencing and other high-throughput experimental technologies. We used KOBAS software for the statistical improvement of differentially expressed genes in KEGG pathways.

### Criteria for screening candidate genes through pathway analysis

To achieve information with respect to metabolism and signal transduction of phyto-hormones, the transcripts that are involved in these pathways were generally filtered by relating annotation of KEGG and KO ID in KEGG maps. Though, the annotations of a solitary transcript from Nr, Swiss-Prot and KEGG databases were not constantly dependable with another. Hence, additional stringent screening conditions were set up to guarantee that the annotation were consistent in at least two distinct databases. A few sequences with uncertain annotation were established online by Nr association using BLASTX. To compare the homologs with the other species in the Asteraceae, we executed screening parameters as E-value ≤ -5 using local BLASTX.

### Real-time quantitative PCR analysis of filtered genes

As the transcriptome was the pooled information with the various libraries, qRT-PCR was performed to identify the expression of particular genes at particular stages. This was accomplished for certain gerbera genes that were up and down-regulated in healthy and diseased samples. Gerbera plants were grown in growth chamber in Fujian Agriculture and Forestry University, in full-strength Murashige and Skoog solution. The plants were inoculated with *Phytophothora cryptogea* a root-rot pathogen. Leaf, petiole and root samples of healthy plant and diseased plants were collected. After the pathogen inoculation, leaf, petiole and root samples were collected after two days, four days and six days of infection. These healthy and diseased (leaf, petiole, and root) samples were used for qRT-PCR analyses. Total RNA of three biological replicates of root, petiole, and leaf samples from control plants and *Phytophothora cryptogea* infected plants were extracted using the same method as mentioned before. A 2-μg sample of entire RNA after DNase action was used for cDNA synthesis by using cDNA synthesis kit. The relative quantitative study was accomplished under the following conditions: 95°C for 30 s and 40 cycles at 95°C 30 s, 60°C 25 s. A melting curve analysis extending from 60 to 95°C was used to distinguish different amplicons. Three specialized replicates inside each biological replicate were used for individually tested sample and template free negative controls. The candidate genes with length more than 500 bp were primarily screened for designing primers. The gene specific primer sets in the particular sequences were designed using DNAMAN 6.0 software (S4 Table). The 18S was used as housekeeping gene for standardization. Three biological replicates were analyzed for each gene. The average threshold cycle (Ct) was calculated and relative gene expression was determined using the 2^-ΔΔCT^ method [77]. A one-way ANOVA analysis of the gene expression level of the samples at different days after inoculation was performed using the software Statistical Package for Social Science (IBM. SPSS 22). The individual treatment means were compared using the LSD (least significance difference) test.

### Conclusion

RNA-Seq analysis uncovered the genes and network involved in gerbera disease resistance. We analyzed the expression summaries of *Gerbera hybrida* inoculated with root rot pathogenic/non-pathogenic fungal isolates. A noticeable differential responsive expression pattern in host-pathogen combination was observed. Progressively, active, and drastic responses were observed in response to root-rot pathogen infection. These responses include a stronger activation of several well-known defense related genes and genes involved in Tyr metabolism, plant hormone signal transduction and catalytic activity. GO and KEGG pathway analysis identified various molecular mechanisms that intricate in disease resistance and corresponding infection related pathways. This information is valuable for further genomic studies in Asteraceae and this can be utilized as a reference for other closely related species having ornamental importance. The outcomes of this research are useful to understand the molecular basis of gerbera root rot associations and new resistance mechanisms in gerbera against *Phytophothora cryptogea*.

## Supporting information

S1 Fig Length distribution of transcript and unigenes in gerbera. (TIF)

S2 Fig GO classification of 294521 unigenes in gerbera. (TIF)

S3 Fig KOG classification of 15528 unigenes in gerbera. (TIF)

S4 Fig KEGG classification of 14335 unigenes in gerbera. (TIF)

S5 Fig Most enriched up and down regulated DEGs in Dis vs Ck in gerbera. (TIF)

S1 Table Annotation summary of unigenes in gerbera. (XLSX)

S2 Table Up and down regulated DEGs related to healthy and root-rot disease gerbera plant. (XLSX)

S3 Table Top 17 KEGG pathways of DEGs in healthy and diseased gerbera plant. (XLSX)

S4 Table List of primers used in this research. (XLSX)
