# Supplementary figures and images for: RNA-Seq analysis reveals an essential role of tyrosine metabolism pathway in response to root-rot infection in Gerbera hybrida
Source: PLoS One. 2019 Oct 23;14(10):e0223519. doi: 10.1371/journal.pone.0223519 (PMC6808435; doi:10.1371/journal.pone.0223519)

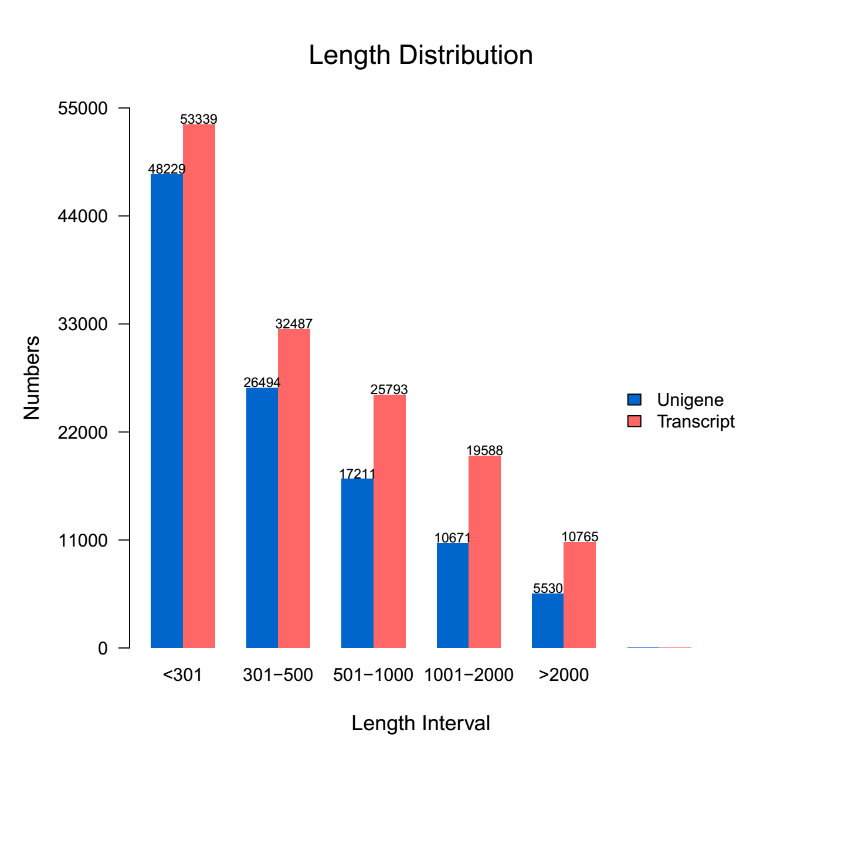

Supplement: S1 Fig — (TIF) [file pone.0223519.s001.tif]

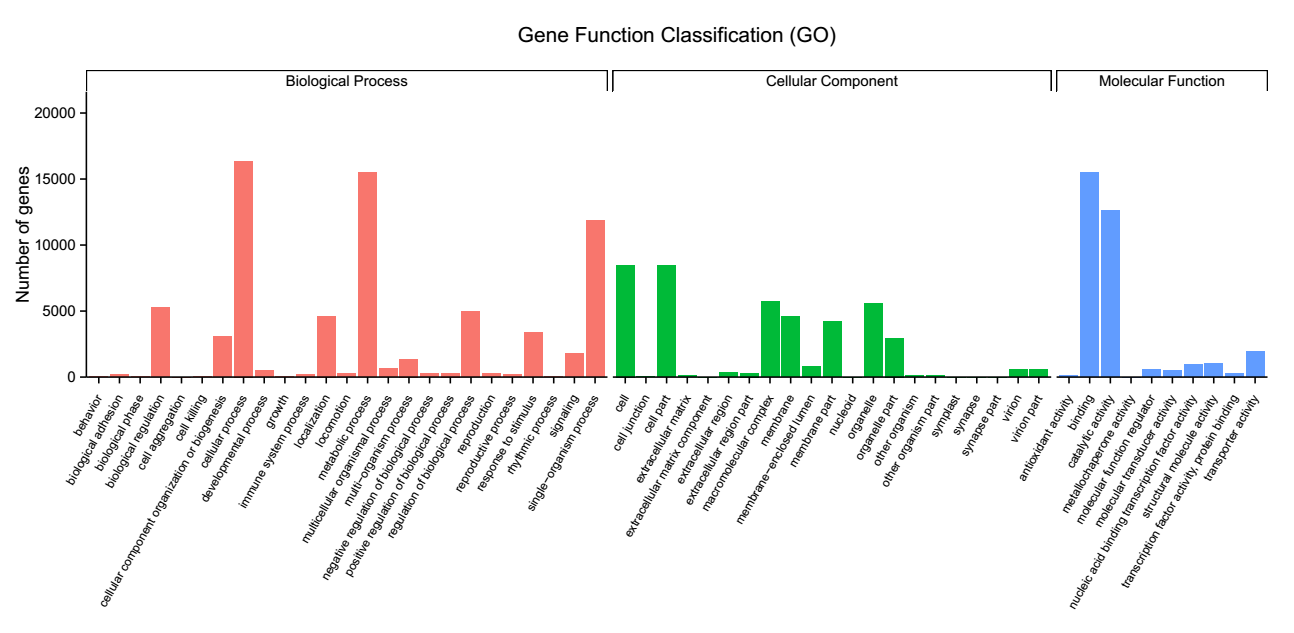

Supplement: S2 Fig — (TIF) [file pone.0223519.s002.tif]

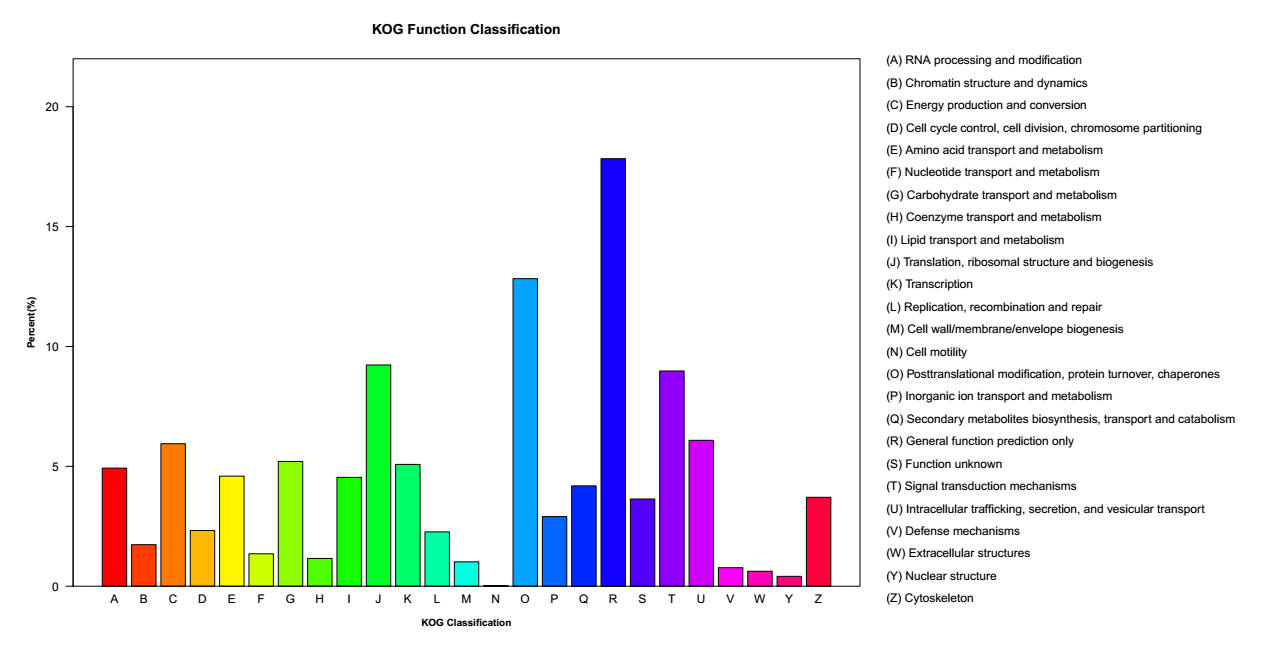

Supplement: S3 Fig — (TIF) [file pone.0223519.s003.tif]

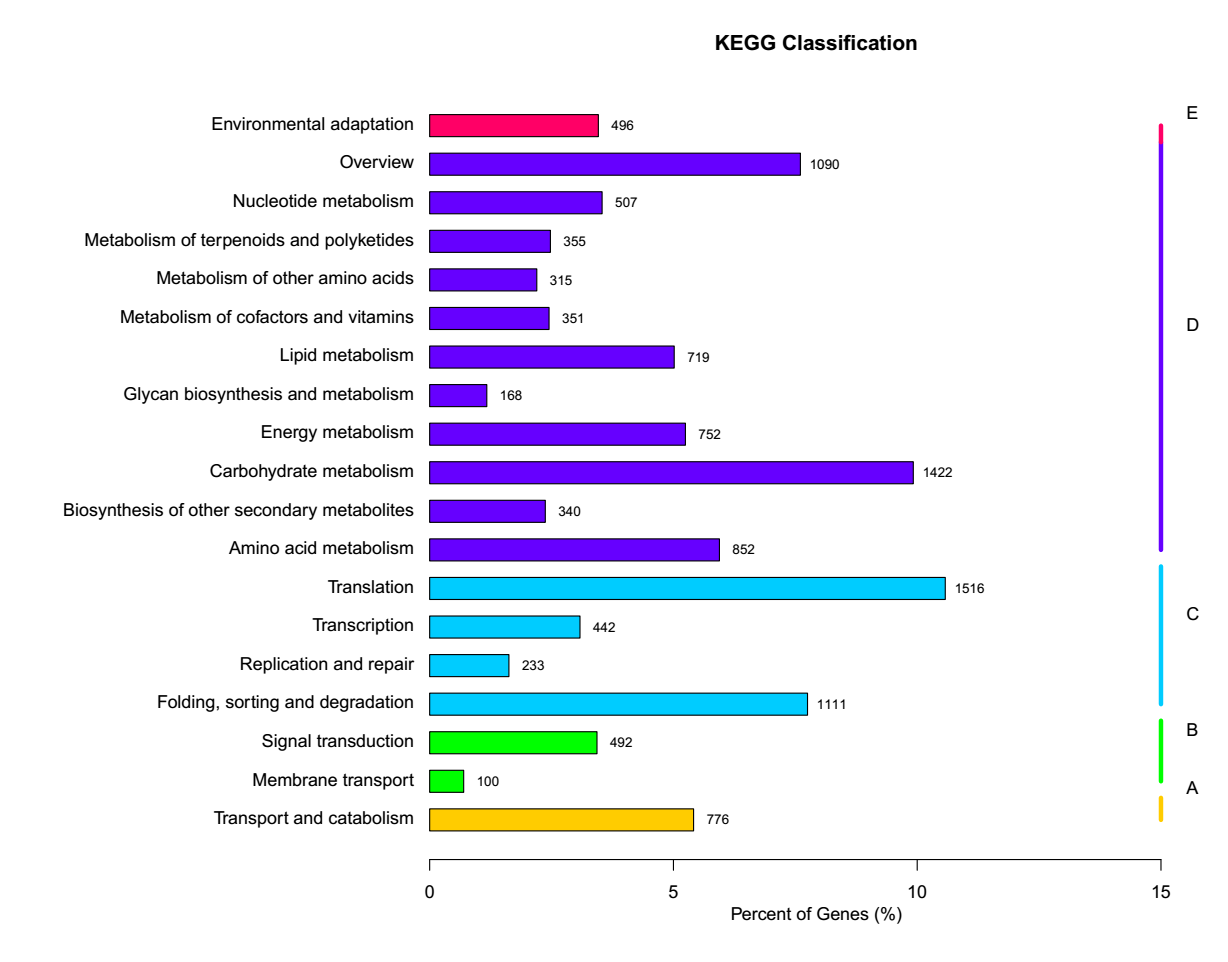

Supplement: S4 Fig — (TIF) [file pone.0223519.s004.tif]

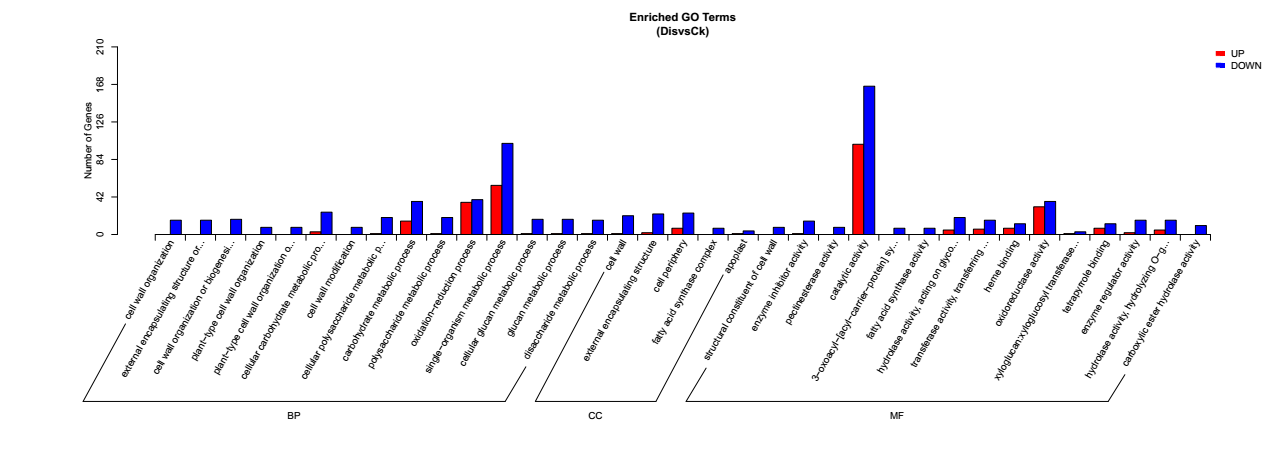

Supplement: S5 Fig — (TIF) [file pone.0223519.s005.tif]
